# From Glucose to Lactate and Transiting Intermediates Through Mitochondria, Bypassing Pyruvate Kinase: Considerations for Cells Exhibiting Dimeric PKM2 or Otherwise Inhibited Kinase Activity

**DOI:** 10.3389/fphys.2020.543564

**Published:** 2020-12-01

**Authors:** Christos Chinopoulos

**Affiliations:** Department of Medical Biochemistry, Semmelweis University, Budapest, Hungary

**Keywords:** cancer, glycolysis, mitochondria, metabolomics, Warburg effect, oncometabolism, lactate dehydrogenase

## Abstract

A metabolic hallmark of many cancers is the increase in glucose consumption coupled to excessive lactate production. Mindful that L-lactate originates only from pyruvate, the question arises as to how can this be sustained in those tissues where pyruvate kinase activity is reduced due to dimerization of PKM2 isoform or inhibited by oxidative/nitrosative stress, posttranslational modifications or mutations, all widely reported findings in the very same cells. Hereby 17 pathways connecting glucose to lactate bypassing pyruvate kinase are reviewed, some of which transit through the mitochondrial matrix. An additional 69 converging pathways leading to pyruvate and lactate, but not commencing from glucose, are also examined. The minor production of pyruvate and lactate by glutaminolysis is scrutinized separately. The present review aims to highlight the ways through which L-lactate can still be produced from pyruvate using carbon atoms originating from glucose or other substrates in cells with kinetically impaired pyruvate kinase and underscore the importance of mitochondria in cancer metabolism irrespective of oxidative phosphorylation.

## Glucose and Lactate in Cancer: Background

**FIGURE 1:**
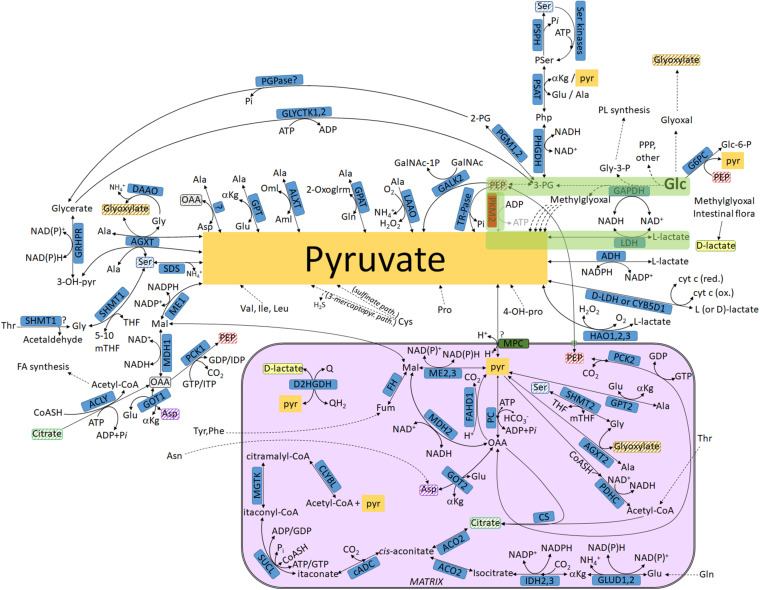
Biochemical pathways connecting glucose or other metabolites to pyruvate and L- or D-lactate. The box in magenta represents a mitochondrion. Glycolysis is highlighted in green. Metabolites found both inside and outside the mitochondria that are not connected with an arrow are highlighted in matching striped colors (to avoid arrow clutter). For abbreviations, see Table 1.

It is a well-known fact that most cancers exhibit increased rates in glucose consumption (Bose and Le, 2018). This is clinically exploited by following radionuclide-labeled glucose analogs for the purpose of tumor imaging in living human beings (Feng et al., 2019). The very same cancers are also known to be major lactate producers, which is important for their survival (de la Cruz-Lopez et al., 2019). The combination of an increased consumption of glucose with an increase in lactate output led to the assumption that cancers exhibit an increase in glycolysis; although this is true, serving the purpose of generating glycolytic metabolites which are diverted toward biosynthetic processes (DeBerardinis et al., 2008) and NADPH by the pentose phosphate pathway (Icard and Lincet, 2012), most tumors express a dimeric form of the M2 isoform of pyruvate kinase which has been reported to be much less active than that found in healthy cells; furthermore, numerous posttranslational modifications and mutations have been reported for this gene product, leading to a much reduced activity but still fueling cancer aggression (see section “Pyruvate Kinase”). Even more so, tumor cells with undetectable levels of pyruvate kinase still producing lactate can be found *in vivo* (Israelsen et al., 2013). On one hand, the decrease in pyruvate kinase activity is important for maintaining a metabolite “traffic jam,” forcing upstream metabolites toward biosynthetic pathways; on the other hand, it points to a metabolic conundrum because L-lactate may only originate from pyruvate, a metabolite arising from phosphoenolpyruvate (PEP) through pyruvate kinase in glycolysis (see Figure 1). The purpose of this review is to highlight the pathways that can lead to pyruvate and lactate—even commencing from glucose—bypassing pyruvate kinase. This is important because (i) carbon-labeled atoms in glucose may appear in lactate without net ATP production from glycolysis and (ii) hints on the possibility that other pathways leading to pyruvate/lactate could be crucial for cancer cell survival that are perhaps amenable to pharmacological and/or genetic manipulation. The list of pathways appearing below has been assembled by mining the following databases: Kyoto Encyclopedia of Genes and Genomes^1^ (Kanehisa and Goto, 2000), BRaunschweig ENzyme Database^2^ (Jeske et al., 2019), Metabolic Atlas^3^ (Robinson et al., 2020), Biochemical, Genetic, and Genomic knowledge base^4^ (King et al., 2016), MetaNetX^5^ (Moretti et al., 2016), Human Metabolome Database^6^ (Wishart et al., 2018), and Virtual Metabolic Human^7^ (Noronha et al., 2019).

## Pyruvate Kinase

Pyruvate kinase generates ATP at the “substrate level” in the absence of oxygen by catalyzing the dephosphorylation of PEP to pyruvate (see Figure 1). There are four isoforms denoted as L, R, M1, and M2. For details regarding kinetic properties, tissue distribution, and regulation, the reader is referred to the review by Israelsen and Vander Heiden (2015). In the present review, the PKM2 isoform will be specifically examined; for a more thorough evaluation, the reader is referred to Li et al. (2014, 2018), Wong et al. (2015); Yang and Lu (2015), Dayton et al. (2016b), Hsu and Hung (2018), and Alquraishi et al. (2019). The non-enzymatic functions of PKM2 are examined elsewhere (Hoshino et al., 2007; Stetak et al., 2007; Luo et al., 2011; Yang et al., 2012; Yang and Lu, 2013).

Basically, PKM2 exhibits lower enzymatic activity compared to that by PKM1 (Yamada and Noguchi, 1999) and is allosterically regulated by fructose-1,6-bisphosphate (FBP); it exists either as a dimer with low affinity for PEP or as an FBP-bound tetramer with high affinity for PEP (Mazurek et al., 2005; Zhang et al., 2019). Although PKM2 has been branded as “the predominant isoform in cancer cells” (Altenberg and Greulich, 2004; Mazurek et al., 2005), further scrutiny in 25 human malignant cancers, six benign oncocytomas, tissue-matched controls, and several cell lines showed that “PKM2 dominance was not a result of a change in isoform expression, since PKM2 was also the predominant PKM isoform in matched control tissues.” Therefore, a switch from PKM1 to PKM2 isoform expression during malignant transformation may not be taking place, as previously postulated (Christofk et al., 2008). Mindful of the controversy surrounding the proposed functions of PKM2 (Hosios et al., 2015; Harris and Fenton, 2019), the group of Vander Heiden characterized the effects of cancer−associated PKM2 mutations on enzyme kinetics and allosteric regulation and reported that a decrease in PKM2 activity supports the rapid proliferation of cells (Liu V. M. et al., 2020). This is in line with earlier reports showing that a decrease in PKM2 activity due to posttranslational modifications (Lv et al., 2011) or inhibition by oxidative stress (Anastasiou et al., 2011) promotes tumor growth (Prakasam et al., 2018). Alternatively, exposure to small molecule PKM2 activators or expression of the constitutively active PKM1 thwarts cancer cell proliferation (Anastasiou et al., 2012). Finally, it has been also shown that PKM2 is not even required for the growth of many cancers (Cortes-Cros et al., 2013; Israelsen et al., 2013; Wang et al., 2014; Lunt et al., 2015; Dayton et al., 2016a, 2018; Lau et al., 2017; Tech et al., 2017; Hillis et al., 2018). In aggregate, the consensus seems to be that the lower the pyruvate kinase activity, the greater the stimulation of tumor growth. As discussed in the section below entitled “Evidence Showing That Pyruvate Kinase Inhibition Does Not Lead to a Proportional Decrease in Pyruvate/Lactate Formation,” even those cells exhibiting low—or even undetectable—pyruvate kinase activity still produce lactate, which begs the question: where does this lactate come from?

## Evidence Showing That Pyruvate Kinase Inhibition Does Not Lead to a Proportional Decrease in Pyruvate/Lactate Formation

In Cortes-Cros et al. (2013), it was shown that knockdown of both PKM1 and PKM2 (PKM2 knockdown was on the order of > 95%) leading to an approximately fivefold decrease in overall pyruvate kinase activity yielded only a ∼50% decrease in the appearance of ^13^C originating from glucose to lactate.

In Chaneton et al. (2012), silencing of both PKM1 and PKM2 to an extent greater than 90% led to only a ∼30% decrease in pyruvate and lactate production, while PEP concentration increased by 100%.

In Vander Heiden et al. (2010), it was shown that cancer cell lysates expressing no pyruvate kinase activity produced 50% of pyruvate from PEP compared with the total cell lysates. Although in this work it was postulated that phosphate from PEP is transferred to the catalytic histidine on human PGAM1, this claim was subsequently rejected by the same authors, attributing their earlier findings to contaminating ATP−dependent protein kinases (Hosios et al., 2015).

In all of the abovementioned studies, it was assumed that, in view of severely diminished pyruvate kinase activity, pyruvate and lactate production is attributed to carbon sources other than glucose. Indeed Yu et al. (2019), determined that, in pancreatic ductal adenocarcinoma cells with PKM1 and PKM2 knockdown, cysteine catabolism generated ∼20% of intracellular pyruvate. The purpose of the present review is to not only outline these pathways but also show additional ways for obtaining ^13^C labeling in pyruvate or lactate originating from glucose; furthermore, since some of these pathways involve intermediates that transit through the matrix, the role of the mitochondria is emphasized, which is unrelated to the concept of oxidative phosphorylation.

## Pathways Leading to Pyruvate Commencing From Glucose: Intermediates Not Transiting Through the Mitochondria

**FIGURE 2:**
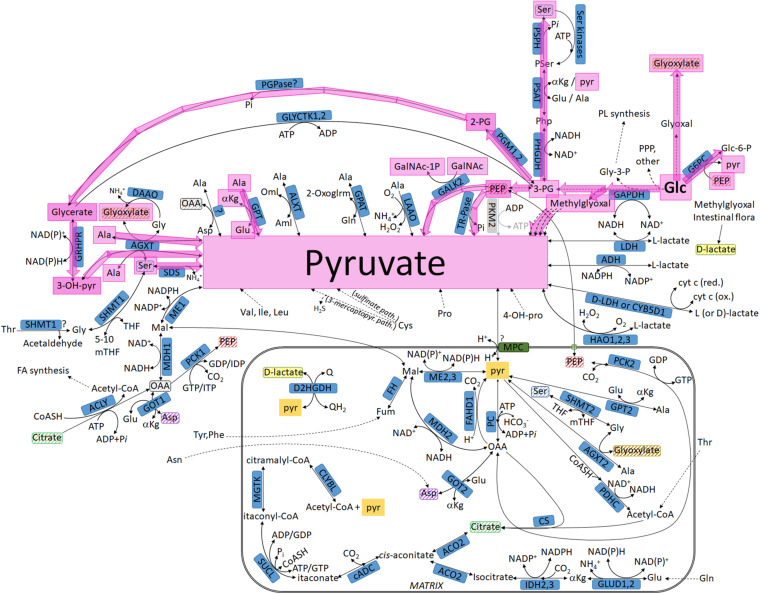
Pathways leading to pyruvate commencing from glucose, highlighted in lavender: intermediates not transiting through the mitochondria. For abbreviations, see Table 1.

The pathways shown in this section refer to Figure 2 (lavender arrows). Multiple arrows imply multiple biochemical steps.

(1) Glc + PEP → Glc-6-P + pyruvate: This reaction is catalyzed by glucose-6-phosphatase (G6PC) (Nordlie, 1974; Colilla et al., 1975) (for abbreviations, see Table 1). In humans, G6PC expression was reported to be elevated in GBM when compared with normal brain (Abbadi et al., 2014), while in rodent hepatomas it was found to be decreased (Weber and Cantero, 1955).

**TABLE 1:** Abbreviations.

2-Oxoglrm	2-oxoglutaramate (a-ketoglutaramate)
2-PG	2-Phosphoglycerate
3-OH-pyr	3-hydroxypyruvate
3-PG	3-Phosphoglycerate
4-OH-proline	4-hydroxyproline
5-10 mTHF	5-10 methylene-Tetrahydrofolate
ACLY	ATP Citrate Lyase
ACO	Aconitase
ADH	Alcohol Dehydrogenase
AGXT	Alanine-glyoxylate Aminotransferase
aKG	a-ketoglutarate
Ala	Alanine
ALXT	Alanine-Ketomalonate Transaminase
Aml	Aminomalonate
Asn	Asparagine
Asp	Aspartate
cADC	cis-Aconitate Decarboxylase
CLYBL	Citramalyl-CoA Lyase
CS	Citrate Synthase
CYB5D1	Cytochrome B5 Domain-Containing Protein 1
Cys	Cysteine
D2HGDH	D-2-Hydroxyglutarate Dehydrogenase
DAAO	D-amino acid Oxidase
D-LDH	D-Lactate Dehydrogenase
FAHD	Acylpyruvase
FH	Fumarate Hydratase
Fum	Fumarate
G6PC	Glucose 6 phosphatase
GALK	N-acetylgalactosamine Kinase
GalNAc	N-Acetylgalactosamine
GalNAc-1-P	N-Acetylgalactosamine-1-Phosphate
GAPDH	Glyceraldehyde 3 Phosphate Dehydrogenase
Glc	Glucose
Glc-6-P	Glucose-6-phosphate
Gln	Glutamine
Glu	Glutamate
GLUD	Glutamate Dehydrogenase
Gly	Glycine
Gly-3-P	Glyceraldehyde-3-Phosphate
GLYCTK	Glycerate Kinase
GOT	Aspartate Aminotransferase
GPAT	Glutamine-Pyruvate Transaminase
GPT	Alanine Aminotransferase
GRHPR	Glyoxylate Reductase
HAO	Hydroxyacid Oxidase
IDH	Isocitrate Dehydrogenase
Ile	Isoleucine
KGDHC	a-Ketoglutarate Dehydrogenase Complex
LAAO	L-amino-acid Oxidase
LDH	Lactate Dehydrogenase
Leu	Leucine
Mal	Malate
MDH	Malate Dehydrogenase
ME	Malic Enzyme
MGTK	Methylglutaconase
MPC	Mitochondrial Pyruvate Carrier
mTHF	methyl-Tetrahydrofolate
OAA	Oxaloacetate
Oml	Oxomalonate
PCK	Phosphoenolpyruvate Carboxykinase
PCK	Pyruvate Carboxylase
PDHC	Pyruvate Dehydrogenase Complex
PEP	Phosphoenolpyruvate
PGM	Phosphoglucomutase
PGPase	2-phosphoglyceric acid Phosphatase
Phe	Phenylalanine
PHGDH	Phosphoglycerate Dehydrogenase
Php	Phosphohydroxypyruvate
PKM2	Pyruvate Kinase isoform M2
PL	Phospholipids
PPP	Pentose Phosphate Pathway
PSAT	Phosphoserine Aminotransferase
Pser	Phosphoserine
PSPH	Phosphoserine Phosphatase
pyr	Pyruvate
Q	Quinone
QH2	Quinol
SDH	Succinate Dehydrogenase
SDS	Serine Dehydratase
Ser	Serine
SHMT	Serine Hydroxymethyltransferase
SUCL	Succinate-CoA Ligase
THF	Tetrahydrofolate
Thr	Threonine
TR-Pase	Tartrate-resistant acid Phosphatase
Tyr	Tyrosine
Val	Valine

(2) Glc →→→ methylglyoxal →→→ pyruvate: This may occur through four different routes involving aldehyde dehydrogenase 9, zinc binding alcohol dehydrogenase domain containing two [more recently renamed to prostaglandin reductase 3 (Yu et al., 2013)] and at least two oxoaldehyde dehydrogenases; for details, see Vander Jagt and Hunsaker (2003). Methylglyoxal has been reported to trigger metastasis in breast, anaplastic thyroid, and colorectal cancer (Chiavarina et al., 2017; Antognelli et al., 2019; Nokin et al., 2019).

(3) Glc →→→ PEP → pyruvate: the terminal reaction is catalyzed by tartrate-resistant acid phosphatases (TRAP), the molecular identity of which remained unknown well after their biochemical characterization (Helwig et al., 1978; Chen and Chen, 1988; Hayman et al., 1989); they are most likely substantiated by a metalloprotein enzyme with the ability to catalyze the hydrolysis of orthophosphate monoesters under acidic conditions (Bull et al., 2002). The expression of this enzyme (TRAP) is a marker of bone disease in cancer patients (Nguyen et al., 1991; Koizumi and Ogata, 2002; Mose et al., 2003; Terpos et al., 2003; Chao et al., 2005).

(4) Glc →→→ PEP; PEP + GalNAc → GalNAc-1P + pyruvate: Terminal reaction catalyzed by N-acetylgalactosamine kinase isoforms 1 or 2 (Pastuszak et al., 1996). These enzymes are implicated in many signaling pathways inherent to carcinogenesis (Zeidan and Hart, 2010).

(5) Glc →→→ 3-PG → 2-PG (by phosphoglucomutase 1 or 2) → glycerate [probably through 2-phosphoglyceric acid phosphatase (Baranowski et al., 1968)] → 3-OH-pyr [by glyoxylate reductase (Mdluli et al., 2005)]; 3-OH-pyr + Ala (or glyoxylate) → Gly + pyruvate (or Ser): the terminal reaction is catalyzed by alanine-glyoxylate aminotransferase (Danpure et al., 2003). The mitochondrial isoform of the latter enzyme (alanine-glyoxylate aminotransferase isoform 2, AGXT2) has been reported to form glycine and pyruvate from alanine and glyoxylate; this reaction has been confirmed in normal tissues (Holmes and Assimos, 1998) and HepG2 cancer cells (Baker et al., 2004). The same reaction has been reported to take place in peroxisomes (Poore et al., 1997). On the other hand, loss of alanine-glyoxylate aminotransferase (AGXT) expression has been reported to accelerate the progression of hepatocellular carcinoma (Sun et al., 2019). A “futile cycle” may exist between 3-PG and glycerate through 2-phosphoglyceric acid phosphatase and glycerate kinase 1 and 2; glycerate kinase 2 is also found in the mitochondria (Guo et al., 2006).

(6) Glc →→→ 3-PG → phosphohydroxypyruvate (Php), catalyzed by phosphoglycerate dehydrogenase; Php + Ala → phosphoserine (Pser) + pyruvate, catalyzed by phosphoserine aminotransferase (PSAT) (Hirsch and Greenberg, 1967): PSAT overexpression is associated with increased tumorigenicity in human esophageal squamous cell carcinoma (Liu et al., 2016) and colon carcinomas (Yoon et al., 2015) and a poor outcome on tamoxifen therapy in recurrent breast cancer (De Marchi et al., 2017); conversely, its selective loss suppresses migration, invasion, and experimental metastasis in triple negative breast cancer (Metcalf et al., 2020).

(7) Glc →→→ 3-PG →Php (catalyzed by phosphoglycerate dehydrogenase); Php + Ala (or Glu) → Pser + pyruvate (or →Kg); the latter reaction is catalyzed by phosphoserine aminotransferase; Pser → Ser → pyruvate, catalyzed by serine dehydratase (Ogawa et al., 2006) or serine dehydratase-like (SDSL) (Ogawa et al., 2006). Notably, SDS was reported to be absent from human colon carcinomas (Snell et al., 1988).

(8) Glc →→→ Glyoxal →→→ glyoxylate (Lange et al., 2012); glyoxylate + 3-OH-pyr (or Ala) → Gly + pyruvate (or Ser): the terminal reaction is catalyzed by AGXT (for considerations related to cancer, see pathway no. 5).

(9) Glc →→→ 3-PG →Php (catalyzed by phosphoglycerate dehydrogenase); Php + Glu → Pser + →Kg; latter reaction catalyzed by phosphoserine aminotransferase; →Kg + Ala → Glu + pyruvate, catalyzed by alanine aminotransferase (GPT; for considerations related to cancer, see pathway no. 6).

## Pathways Leading to Pyruvate Commencing From Glucose: Intermediates Transiting Through the Mitochondria

**FIGURE 3:**
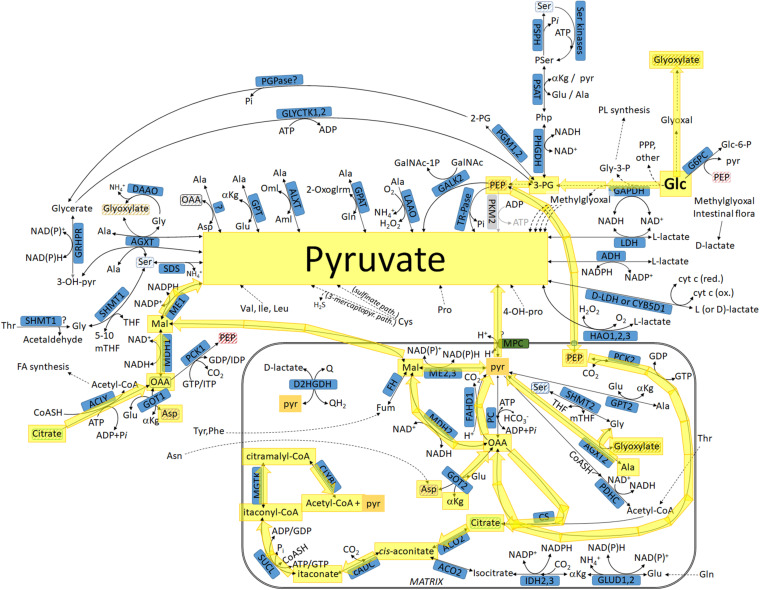
Pathways leading to pyruvate commencing from glucose, highlighted in yellow: intermediates transiting through the mitochondria. For abbreviations, see Table 1.

These pathways depend on one or more of three critical parameters: (1) glyoxylate entry into the mitochondria, (2) reversibility of the matrix phosphoenolpyruvate carboxykinase (PCK2), and (3) reversibility of the mitochondrial pyruvate carrier (MPC). Regarding glyoxylate, I was unable to find information on its transport across the inner mitochondrial membrane; however, it is known that it can be processed by the matrix-localized AGXT2 (Kakimoto et al., 1969). PCK2 expression and activity level are critical for many cancer types: in tumor-initiating enriched prostate cancer cell clones, PCK2 was overexpressed, and this correlated with more aggressive tumors and lower survival rates (Zhao et al., 2017); in lung cancer cell lines and in non-small cell lung cancer samples, PCK2 expression and activity were enhanced under low-glucose conditions (Leithner et al., 2015); finally, it was reported that PCK2 is required for glucose-independent cancer cell proliferation and tumor growth *in vivo* (Vincent et al., 2015). Regarding PCK2 reversibility, the enzyme has been shown to operate in the reaction toward OAA synthesis in mitochondria from rabbit liver (Carlsen et al., 1988), pigeon and rat liver (Wiese et al., 1996), guinea pig liver (Garber and Ballard, 1970; Garber and Salganicoff, 1973), rabbit enterocytes (Wuensch and Ray, 1997), chicken liver (Hebda and Nowak, 1982; Makinen and Nowak, 1983; Wilson et al., 1983; Erecinska and Wilson, 1984), and bullfrog liver (Goto et al., 1980). However, in Vincent et al. (2015), it was shown that a fraction of pyruvate originated from glutamine from PEP through PCK2. With respect to the reversibility of the MPC, this is a working hypothesis because there are no data showing pyruvate release from normally polarized mitochondria. Nevertheless, this is not a far-fetched hypothesis: succinate and other metabolites are effluxed from the mitochondria for non-metabolic roles against a hyperpolarized membrane potential (Mills et al., 2016), demonstrating that this is possible under appropriate conditions. It may be also relevant that pyruvate catabolism through the pyruvate dehydrogenase complex is associated with suppression of tumor growth *in vitro* and *in vivo* (Michelakis et al., 2008); relevant to this, genes coding for both the pyruvate dehydrogenase complex and pyruvate carboxylase in certain cancers are usually downregulated (Yuen et al., 2016); furthermore, pyruvate is found in blood plasma, urine, and cerebrospinal fluid, and its presence there is not associated with damage of plasma membranes. Of course, this does not mean that extracellular pyruvate originated from the mitochondria, but it indicates that it can cross the plasma membrane through monocarboxylate transporters, some of which are distributed both in plasma and in the inner mitochondrial membrane (Hussien and Brooks, 2011); indeed monocarboxylate transporter 1, which is one of the four known pyruvate transport mechanisms, was recently shown to export pyruvate from the cell (Hong et al., 2016); however, mitochondrial pyruvate export remains hypothetical especially in view of the fact that its exit is influenced by the membrane potential and →pH. It was also recently reported that loss of an MPC isoform prior to a tumorigenic stimulus doubled the frequency of adenoma formation and produced higher-grade tumors, and this was associated with a glycolytic metabolic phenotype and increased expression of stem cell markers (Bensard et al., 2020). Mindful of the above, these pathways are as shown in Figure 3 (yellow arrows).

(10) Glc →→→ glyoxal →→→ glyoxylate: Glyoxylate enters the mitochondria; glyoxylate + Ala → Gly + pyruvate through AGXT2. Pyruvate may exit the mitochondria through the MPC (for considerations related to cancer, see pathway no. 5).

(11) Glc →→→ PEP which enters the mitochondria; PEP transport across the inner membrane of mammalian mitochondria has been demonstrated to occur by the tricarboxylate carrier by Robinson (1971) and the group of Soling et al. (1971) and Kleineke et al. (1973) and to a lesser extent by the adenine nucleotide carrier, shown by the Shug and Shrago (1973); Sul et al. (1976) and in Drahota et al. (1983) and reviewed in Passarella et al. (2003). The possibility of a PEP/pyruvate transporter has also been put forward (Satrustegui et al.,2007). More recently, PEP cycling *via* mitochondrial PEPCK evoking PEP transport across the inner mitochondrial membrane has also been demonstrated by the group of Kibbey (Stark et al., 2009); PEP → OAA by PCK2; OAA → pyruvate by reverse operation of PC. However, this is expected to be a very minor path. Pyruvate may exit the mitochondria through the MPC.

(12) Glc →→→ PEP; PEP enters the mitochondria through the means outlined in pathway 11. PEP → OAA by PCK2; OAA → pyruvate by FAHD1 (Pircher et al., 2011, 2015). FAHD1 also converts 3-acylpyruvate, acetylpyruvate, and fumarylpyruvate to pyruvate (Pircher et al., 2011). It is not known where acetylpyruvate comes from, but its existence is known since Krebs reported it (Krebs and Johnson, 1937). Pyruvate may exit the mitochondria through the MPC. FAHD1 depletion has been shown to induce premature senescence in human endothelial cells by inhibiting mitochondrial metabolism (Petit et al., 2017); however, this might be a double-edged sword since OXPHOS capacity has been inversely correlated with malignancy in several cell types (Zhou et al., 2003; Matoba et al., 2006; Hu et al., 2012; Hall et al., 2013; Bartesaghi et al., 2015; Nicolay et al., 2015; Capala et al., 2016; Smith et al., 2020).

(13) Glc →→→ PEP; PEP enters the mitochondria through the means outlined in pathway 11; PEP → OAA by PCK2; OAA → Mal by MDH2; Mal → pyruvate by ME2,3 (Zelewski and Swierczynski, 1991). Pyruvate may exit the mitochondria through the MPC. *ME2* knockdown suppresses tumor growth in lung cancer (Ren et al., 2014), while *ME2,3* deletions confer lethality in pancreatic cancer (Dey et al., 2017).

(14) Glc →→→ PEP; PEP enters the mitochondria through the means outlined in pathway 11; PEP → OAA by PCK2; OAA → Mal by MDH2; Mal exits the mitochondria; Mal → pyruvate by ME1 (Zelewski and Swierczynski, 1991; Loeber et al., 1994). *ME1* knockdown inhibits the growth of colon cancer cells (Murai et al., 2017), and its overexpression is associated with larger breast tumor size, higher incidence of lymph node metastasis, and higher incidence of lymph–vascular invasion (Liu C. et al., 2020). In the same line, ME1 is associated with tumor budding—a phenomenon representing epithelial to mesenchymal transition—in oral squamous cell carcinomas (Nakashima et al., 2020).

(15) Glc →→→ PEP; PEP enters the mitochondria through the means outlined in pathway 11; PEP → OAA by PCK2; OAA + acetyl-CoA → citrate by CS; citrate exits the mitochondria through the dicarboxylate carrier; citrate + ATP + CoASH → acetyl-coA + ADP + Pi + OAA by ACLY (Chypre et al., 2012); OAA → Mal by MDH1; Mal → pyruvate by ME1 (for considerations related to cancer, see pathway no. 14).

(16) Glc →→→ PEP; PEP enters the mitochondria through the means outlined in pathway 11; PEP → OAA by PCK2; OAA + Glu →→Kg + Asp by GOT2; Asp exits the mitochondria; Asp + →Kg → Glu + OAA by GOT1; OAA → Mal by MDH1; Mal → pyruvate by ME1 (for considerations related to cancer, see pathway no. 14).

(17) Glc →→→ PEP; PEP enters the mitochondria through the means outlined in pathway 11; PEP → OAA by PCK2; OAA + acetyl-CoA → citrate by CS; citrate → cis-aconitate, intermediate of ACO2 reaction; cis-aconitate → itaconate by cADC; itaconate + CoASH + ATP (or GTP) → itaconyl-CoA + Pi + ADP (or GDP) by SUCL (Nemeth et al., 2016); itaconyl-CoA → citramalyl-CoA by methylglutaconase (MGTK); citramalyl-coA → acetyl-CoA + pyruvate by CLYBL (Shen et al., 2017). Pyruvate may exit the mitochondria through the MPC. CLYBL has been reported to be associated with colorectal cancer metastasis (Li and Peng, 2013). Furthermore, *CLYBL* was reported to be overexpressed in 465 out of 38,258 tumor samples in the COSMIC database^8^.

## Pathways Leading to Pyruvate But Not Commencing From Glucose: Intermediates Not Transiting Through the Mitochondria

**FIGURE 4:**
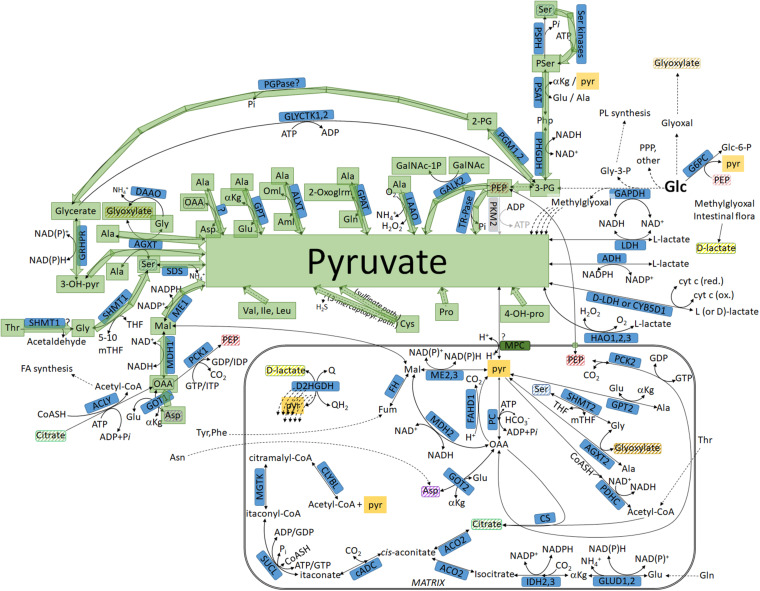
Pathways leading to pyruvate but not commencing from glucose, highlighted in green: intermediates not transiting through the mitochondria. For abbreviations, see Table 1.

These pathways are shown in Figure 4 (green arrows).

(18) Ser → pyruvate, catalyzed by SDS or SDSL (for considerations related to cancer, see pathway no. 6).

(19) Ser →→→ PEP; PEP → pyruvate; terminal reaction catalyzed by tartrate-resistant acid phosphatase (TR-Pases; for considerations related to cancer, see pathway no. 3).

(20) Ser →→→ PEP; PEP + GalNAc → GalNAc-1P + pyruvate. The terminal reaction is catalyzed by N-acetylgalactosamine kinase isoforms 1 or 2 (for considerations related to cancer, see pathway no. 4).

(21) Ala → pyruvate, catalyzed by L-amino-acid oxidases (LAAO) (Nakano et al., 1967): Several mammalian LAAOs have been described, of which the enzyme “interleukin-4 induced gene 1” (IL4I1) is the best characterized (Castellano and Molinier-Frenkel, 2017); IL4I1 expression was reported to be associated with poor prognosis in human breast cancers (Finak et al., 2008).

(22) Ala + 2-oxoglrm → Gln + pyruvate, catalyzed by glutamine-pyruvate transaminase (GPAT) (Cooper and Meister, 1972; Cooper and Kuhara, 2014). GPAT is upregulated in many cancers in a *MYC*-dependent manner (Dong et al., 2020).

(23) Ala + 2-Oml → Aml + pyruvate, catalyzed by alanine-ketomalonate transaminase (ALXT) (Nagayama et al., 1958). I was unable to find relevant literature on ALXT expression or aminomalonate levels and cancer.

(24) Ala + αKg → Glu + pyruvate, catalyzed by GPT: GPT—similar to GPAT—is upregulated in many cancers in a *MYC*-dependent manner (Dong et al., 2020).

(25) Ala + OAA → Asp + pyruvate; enzyme unknown (Rowsell, 1956).

(26) Ala + Glyoxylate → Gly + pyruvate, catalyzed by alanine-glyoxylate aminotransferase (for considerations related to cancer, see pathway no. 5).

(27) Ala + 3-OH-pyr → Ser + pyruvate, catalyzed by alanine-glyoxylate aminotransferase (for considerations related to cancer, see pathway no. 5).

(28) Thr → Gly + acetaldehyde, catalyzed by SHMT1 (Garrow et al., 1993; Pinthong et al., 2014); Gly + 5,10 mTHF → THF + Ser, catalyzed by serine hydroxymethyltransferase 1; Ser → pyruvate, catalyzed by SDS or SDSL. SHMT1 knockdown induces apoptosis in lung cancer cells (Paone et al., 2014), and SHMT inhibitors block the growth of many human cancer cells (Ducker et al., 2017). Patients with high SHMT2 expression exhibit a shorter overall survival rate compared with patients with low expression (Koseki et al., 2018; for further considerations related to SDS or SDSL and cancer, see pathway no. 6).

(29) Asp + αKg → Glu + OAA, catalyzed by GOT1; OAA → Mal by MDH1; Mal → pyruvate by ME1 (for considerations related to cancer, see pathway no. 14).

(30) 4-OH-proline →→→ pyruvate, through glyoxylate formation (see pathway no. 26).

(31) Cys →→→ pyruvate through the sulfinate pathway (Stipanuk, 1979, 2020). Notably, in pancreatic cancer cells exhibiting PKM1/2 knockdown, 20% of intracellular pyruvate originated from cysteine (Yu et al., 2019). The contribution of cysteine catabolism to cancer has been extensively reviewed by Serpa (2020).

(32) Cys → 3-sulfino-L-alanine catalyzed by aspartate 4-decarboxylase (Liu et al., 2012); 3-sulfino-L-alanine is transaminated to 3-sulfinopyruvate by either aspartate aminotransferase or deaminated to the same product by cysteine sulfinic acid deaminase; 3-sulfinopyruvate is non-enzymatically converted to sulfite and pyruvate (Stipanuk, 2020; for considerations related to cancer, see pathway no. 31).

(33) Cys →→→ H_2_S + pyruvate through the 3-mercaptopyruvate pathway (Nagahara and Sawada, 2006). Cys can also transaminate with →-ketoglutarate to form glutamate and 3-mercaptopyruvate though GOT1, exhibiting cysteine transaminase activity. The catabolism of 3-mercaptopyruvate toward pyruvate is outlined in the reactions below (pathway no. 34; for considerations related to cancer, see pathway no. 31).

(34) L-cysteine is isomerized to D-cysteine by cysteine racemase (2-amino-3-mercaptopropionic acid racemase) (Soda and Osumi, 1969); D-Cys is converted to 3-mercaptopyruvate by D-amino acid oxidase and, in turn, to pyruvate and H_2_S by 3-mercaptopyruvate sulfurtransferase (3MST) (Shibuya et al., 2013) or thiosulfate sulfurtransferase (TST) (Pallini et al., 1991). The possibility of conversion of D-Cys to pyruvate by D-cysteine desulfhydrase (Nagasawa et al., 1985) in mammalian cells is yet to be reported. 3-Mercaptopyruvate can also react with hydrogen cyanide, forming pyruvate and thiocyanate in a reaction catalyzed by 3MST or TST; obviously, this is only a very minor route of pyruvate production due to cyanide toxicity (Bhandari et al., 2014; for further considerations related to cancer, see pathway no. 31).

(35) Ser → dehydroalanine (2-aminoacrylate) by serine dehydratase (SDS), serine dehydratase-like protein (SDSL), or serine racemase (SRR): Dehydroalanine can further hydrolyze to NH_3_ and pyruvate through SDS, SDSL, or SRR (Kashii et al., 2005); sometimes this reaction is referred to as hydrolysis by “2-aminoacrylate aminohydrolase.” Dehydroalanine can also spontaneously hydrolyze to NH_3_ and pyruvate through the intermediate 2-iminopropanoate; the latter later part of this spontaneous hydrolysis can be accelerated by 2-iminopropanoate deaminase (Lambrecht et al., 2012). Dehydroalanine can also be derived from 2 3,5-diiodo-L-tyrosine or 3,5-diiodo-L-tyrosine by thyroid peroxidase in the process of forming thyroxine and triiodothyronine, respectively (Gavaret et al., 1980). The crucial importance of serine metabolism for the growth and survival of proliferating cells is extensively reviewed in Yang and Vousden (2016) and Newman and Maddocks (2017).

(36) Se-methyl-L-selenocysteine (SeMSC, Se-methylselenocysteine, methyl selenocysteine) can be deaminated to methaneselenol, NH_3_, and pyruvate by selenocysteine lyase (Esaki et al., 1982). SeMSC can be found in many edible plants, including garlic, onions, and broccoli, as well as in dietary supplements (Yang and Jia, 2014). SeMSC was shown to exhibit anticarcinogenic properties (Ip et al., 1991; Medina et al., 2001) and even potentiate the antitumor activity of anticancer drugs (Cao et al., 2014).

(37) Val →→→ 2-methyl-3-oxopropanoate; 2-methyl-3-oxopropanoate can get transaminated with alanine by AGXT2 to D-3-amino-isobutanoate + pyruvate (Kakimoto et al., 1969). The overexpression of enzymes participating in valine catabolism is associated with poor prognosis in prostate cancer (Mayers et al., 2016) and tumors of the colon (Shan et al., 2019). The role of valine in cancer has been extensively reviewed in Ananieva and Wilkinson (2018) and Lieu et al. (2020).

(38) Leu →→→ 3-methylbutanoyl-CoA; the latter compound is converted to isobutyryl-CoA through branched-chain fatty acid metabolism (many steps); isobutyryl-CoA →→→ 2-methyl-3-oxopropanoate; 2-methyl-3-oxopropanoate can get transaminated with alanine by AGXT2 to D-3-amino-isobutanoate + pyruvate (Kakimoto et al., 1969). Because leucine catabolism shares many steps with that of valine, for considerations related to cancer, see pathway no. 37.

(39) Ile →→→ 2-methylbutanoyl-CoA; the latter compound is converted to isobutyryl-CoA through branched-chain fatty acid metabolism (many steps); isobutyryl-CoA →→→ 2-methyl-3-oxopropanoate; 2-methyl-3-oxopropanoate can get transaminated with alanine by AGXT2 to D-3-amino-isobutanoate + pyruvate (Kakimoto et al., 1969). Because isoleucine catabolism shares many steps with that for valine, for considerations related to cancer, see pathway no. 37.

(40) Pro + αKg + O_2_ → CO_2_ + succinate + trans-4-hydroxy-L-proline, catalyzed by prolyl 4-hydroxylase subunit alpha (isoforms 1, 2, or 3); trans-4-hydroxy-L-proline is then converted to L-1-pyrroline-3-hydroxy-5-carboxylate, also yielding NAD(P)H, by either pyrroline-5-carboxylate reductase (isoforms 1, 2, or 3) or left–right determination factor 1 (LEFTY1), a member of the TGF-→ family of proteins; L-1-pyrroline-3-hydroxy-5-carboxylate can be converted to L-erythro-4-hydroxyglutamate, also yielding NAD(P)H, by aldehyde dehydrogenase 4 family member A1; in turn, L-erythro-4-hydroxyglutamate is transaminated with either OAA by GOT2, yielding 4-hydroxy-2-oxoglutarate + aspartate, or →Kg by GOT1 or GOT2, yielding 4-hydroxy-2-oxoglutarate + glutamate; finally, 4-hydroxy-2-oxoglutarate is converted to glyoxylate and pyruvate by 4-hydroxy-2-oxoglutarate glyoxylate-lyase. It is relevant that increased proline catabolism has been recently reported to support metastasis (Elia et al., 2017). Arg, through either interconversion to metabolites as for proline catabolism or through citrulline/ornithine and the fumarate nucleotide cycle will also lead to pyruvate formation; however, this probably requires inter-organ communication and, thus, may not be found within a single cell. The crucial role of proline catabolism in tumor growth and metastatic progression is extensively reviewed in Phang (2019) and D’Aniello et al. (2020).

## Pathways Leading to Pyruvate But Not Commencing From Glucose: Intermediates Transiting Through the Mitochondria

**FIGURE 5:**
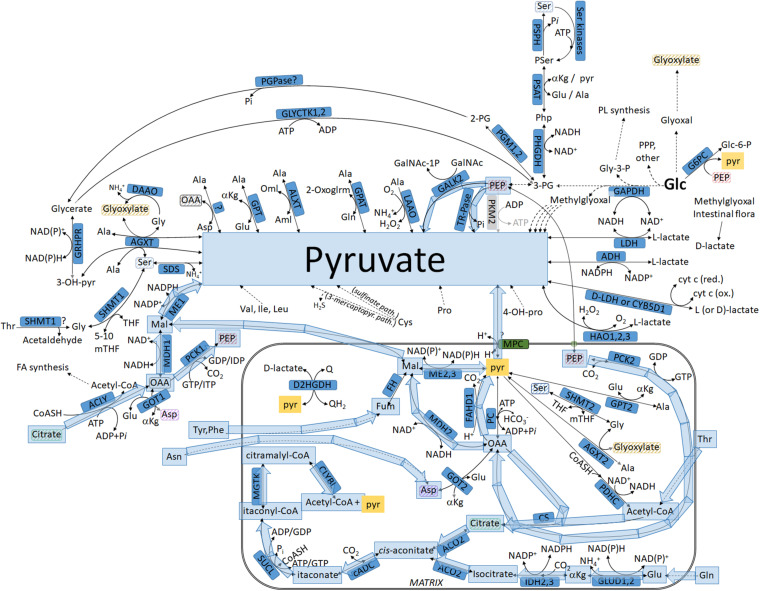
Pathways leading to pyruvate but not commencing from glucose, highlighted in blue: intermediates transiting through the mitochondria. For abbreviations, see Table 1.

These pathways are shown in Figure 5 (blue arrows).

(41) Thr →→→ acetyl-CoA; acetyl-CoA + OAA → citrate, catalyzed by CS; citrate exits the mitochondria through the dicarboxylate carrier; citrate + ATP + CoASH → Acetyl-coA + ADP + Pi + OAA by ACLY (Chypre et al., 2012); OAA → Mal by MDH1; Mal → pyruvate by ME1. The potential role of threonine catabolism in cancer is reviewed in Tsun and Possemato (2015) and Lieu et al. (2020) (for further considerations regarding ME1 and cancer, see pathway no. 14).

(42) Thr →→→ acetyl-CoA; acetyl-CoA + OAA → citrate, catalyzed by CS; citrate → cis-aconitate, intermediate of ACO2 reaction; cis-aconitate → itaconate by cADC; itaconate + CoASH + ATP (or GTP) → itaconyl-CoA + Pi + ADP (or GDP) by SUCL; itaconyl-CoA → citramalyl-CoA by MGTK; citramalyl-coA → acetyl-CoA + pyruvate by CLYBL. Pyruvate may exit the mitochondria through the MPC (regarding threonine and cancer, see pathway no. 41; regarding CLYBL and cancer, see pathway no. 17).

(43) Asn →→→ Asp; Asp + αKg → Glu + OAA by GOT2; OAA by PCK2; OAA → pyruvate by reverse operation of PC. However, this is expected to be a path of a very minor flux. Pyruvate may exit the mitochondria through the MPC. The crucial role of asparagine availability in cancer is explored in Panosyan et al. (2014); Krall et al. (2016), and Knott et al. (2018). However, more emphasis on asparagine availability for anabolic, rather than catabolic, purposes is given.

(44) Asn →→→ Asp; Asp + αKg → Glu + OAA by GOT2; OAA → pyruvate by acylpyruvase (FAHD1). Pyruvate may exit the mitochondria through the MPC (for considerations related to cancer, see pathways no. 12 and 37).

(45) Asn →→→ Asp; Asp + αKg → Glu + OAA by GOT2; OAA → Mal by MDH2; Mal → pyruvate by ME2,3. Pyruvate may exit the mitochondria through the MPC (for considerations related to cancer, see pathways no. 13 and 37).

(46) Asn →→→ Asp; Asp + αKg → Glu + OAA by GOT2; OAA → Mal by MDH2; Mal exits the mitochondria; Mal → pyruvate by ME1 (for considerations related to cancer, see pathways no. 14 and 37).

(47) Tyr, Phe →→→ Fum; Fum → Mal by FH; Mal → pyruvate by ME2,3 (for considerations related to cancer, see pathway no. 13).

(48) Tyr, Phe →→→ Fum; Fum → Mal by FH; Mal exits the mitochondria; Mal → pyruvate by ME1 (for considerations related to cancer, see pathway no. 14).

(49) Tyr, Phe →→→ Fum; Fum → Mal by FH; Mal → OAA by MDH2; OAA → pyruvate by acylpyruvase (FAHD1). Pyruvate may exit the mitochondria through the MPC (for considerations related to cancer, see pathway no. 12).

(50) Thr →→→ acetyl-CoA; acetyl-CoA + OAA → citrate, catalyzed by CS; citrate exits the mitochondria through the dicarboxylate carrier; citrate + ATP + CoASH → acetyl-coA + ADP + Pi + OAA by ACLY; OAA → PEP by PCK1; PEP enters the mitochondria; PEP → OAA by PCK2; OAA → pyruvate by acylpyruvase (FAHD1). Pyruvate may exit the mitochondria through the MPC (for considerations related to cancer, see pathway no. 12).

(51) Thr →→→ acetyl-CoA; acetyl-CoA + OAA → citrate, catalyzed by CS; citrate exits the mitochondria through the dicarboxylate carrier; citrate + ATP + CoASH → acetyl-coA + ADP + Pi + OAA by ACLY; OAA → PEP by PCK1; PEP enters mitochondria; PEP → OAA by PCK2; OAA → Mal by MDH2; Mal → pyruvate by ME2,3. Pyruvate may exit the mitochondria through the MPC (for considerations related to cancer, see pathway no. 13).

(52) Thr →→→ acetyl-CoA; acetyl-CoA + OAA → citrate, catalyzed by CS; citrate exits the mitochondria through the dicarboxylate carrier; citrate + ATP + CoASH → acetyl-CoA + ADP + Pi + OAA by ACLY; OAA → PEP by PCK1; PEP enters the mitochondria; PEP → OAA by PCK2; OAA → Mal by MDH2; Mal exits the mitochondria; Mal → pyruvate by ME1 (for considerations related to cancer, see pathway no. 14).

(53) Thr →→→ acetyl-CoA; acetyl-CoA + OAA → citrate, catalyzed by CS; citrate exits the mitochondria through the dicarboxylate carrier; citrate + ATP + CoASH → Acetyl-coA + ADP + Pi + OAA by ACLY; OAA → PEP by PCK1; PEP + GalNAc → GalNAc-1P + pyruvate. Terminal reaction catalyzed by N-acetylgalactosamine kinase isoforms 1 or 2 (for considerations related to cancer, see pathway no. 4).

(54) Thr →→→ acetyl-CoA; acetyl-CoA + OAA → citrate, catalyzed by CS; citrate exits the mitochondria through the dicarboxylate carrier; citrate + ATP + CoASH → acetyl-coA + ADP + Pi + OAA by ACLY; OAA → PEP by PCK1; PEP → pyruvate; the terminal reaction is catalyzed by tartrate-resistant acid phosphatases (for considerations related to cancer, see pathway no. 3).

## Incompletely Characterized Reactions Forming Pyruvate

In the literature, some reactions have been described to produce pyruvate but are incompletely characterized. These are collectively listed below:

(55) O-carbamoyl-L-serine + H_2_O → pyruvate + 2 NH_3_, catalyzed by carbamoyl-serine ammonia lyase (Copper and Meister, 1973). O-Carbamoyl-L-serine is a weak inhibitor of a phosphate-dependent glutaminase (Shapiro et al., 1979); mindful of the crucial importance of glutamine catabolism through glutaminases in many cancer types, this route of pyruvate provision is probably minor.

(56) L-Cysteine-S-conjugate + H_2_O → a thiol + NH_3_ + pyruvate, catalyzed by cysteine S-conjugate →-lyases (Cooper and Pinto, 2006). The possibility of cysteine S-conjugate β-lyases metabolizing anticancer agents is reviewed in Cooper et al. (2011).

(57) cystathionine + H_2_O → L-homocysteine + pyruvate + NH_3_ or cysteine + H_2_O → sulfide + NH_3_ + pyruvate or cystine → thiocysteine + pyruvate + NH_3_, all catalyzed by cystathionine gamma-lyase (Stipanuk et al., 2006; Chiku et al., 2009). Cystathionine gamma-lyase was reported to be upregulated in bone−metastatic PC3 cells, and its knockdown suppressed tumor growth and metastasis (Wang et al., 2019). In the same line, this enzyme was shown to be upregulated and played a crucial role in the proliferation and migration of breast cancer cells (You et al., 2017).

(58) L-Serine O-sulfate + H_2_O → pyruvate + NH_3_ + sulfate catalyzed by serine-sulfate ammonia-lyase (Tudball and Thomas, 1972). I was unable to find relevant literature on serine-sulfate ammonia-lyase expression or L-serine O-sulfate levels and cancer.

(59) N-Acetylneuraminate → N-acetyl-D-mannosamine + pyruvate catalyzed by N-acetylneuraminate lyase (Brunetti et al., 1962); relevant to this, treatment of HL-60 cells by phorbol esters leads to a marked increase in the activity of this enzyme (Warren, 1986).

(60) D-Alanine + H_2_O + O_2_ → pyruvate + NH_3_ + H_2_O_2_ catalyzed by DAAO (Nagata et al., 1992; Abe et al., 2005; Fuchs et al., 2005; Smith et al., 2009). The interaction of D-alanine (and other D-amino acids) with tumors is reviewed in Bastings et al. (2019).

(61) L-Alanine → pyruvate + NH_3_ catalyzed by glutamate dehydrogenase; this reaction exhibits a weak activity (Silverstein, 1974). The role of glutamate dehydrogenase in cancer cells has been extensively reviewed in Moreno-Sanchez et al. (2020).

(62) 2-Oxosuccinamic acid + Ala → Asn + pyruvate, catalyzed by asparagine aminotransferase (Cooper, 1977; Maul and Schuster, 1986). The origin of 2-oxosuccinamic acid is not known (Cooper et al., 1987). I was unable to find relevant literature on 2-oxosuccinamic acid levels and cancer.

(63) Pyruvate oxime + acetone → pyruvate + acetone oxime, catalyzed by oximinotransferase (Omura et al., 1956). Due to acetone volatility, this is probably a very minor pathway for pyruvate production.

(64) Methylmalonyl-CoA + pyruvate → propionyl-CoA + oxaloacetate catalyzed by methylmalonyl-CoA carboxytransferase (Swick and Wood, 1960). This reaction is reversible and thus may yield pyruvate. I was unable to find relevant literature on methylmalonyl-CoA carboxytransferase and cancer.

(65) L-Alanine + 3-oxopropanoate → pyruvate + →-alanine, catalyzed by either →-alanine-pyruvate transaminase (Ito et al., 2001) or alanine-glyoxylate aminotransferase isoform 2 (Lee et al., 1995) (for considerations related to cancer, see pathway no. 5).

(66) Phenylpyruvate + L-alanine → L-phenylalanine + pyruvate catalyzed by phenylalanine (histidine) transaminase (Minatogawa et al., 1977). Phenylpyruvate has been reported to inhibit pyruvate kinase activity in human brain (Weber, 1969), thus enhancing PK-bypassing pathways. Phenylpyruvate levels were also found to be increased in ovarian cancers (Fong et al., 2011).

(67) 2-Oxoisohexanoate + L-alanine → L-leucine + pyruvate, catalyzed by the mitochondrial branched-chain L-amino acid aminotransferase (Schadewaldt et al., 1995). The role of branched-chain L-amino acid aminotransferase in cancer has been reviewed in Ananieva and Wilkinson (2018).

(68) PCK1, ME1, and ME2,3 may also convert OAA to CO_2_ and pyruvate (Sauer, 1973; Carlson et al., 1978; Bukato et al., 1995; Lee et al., 1995) (for considerations related to cancer, see pathway nos. 13 and 14).

(69) Salsolinol can be converted to salsolinol-1-carboxylate by salsolinol synthetase which can then be catabolized to dopamine and pyruvate (by an unknown enzyme); salsolinol is an endogenous catechol isoquinoline detected in humans derived from dopamine metabolism (Sandler et al., 1973; Collins et al., 1979). Salsolinol has been implicated in the initiation and promotion of alcohol-related breast carcinogenesis (Murata et al., 2016).

## Pathways Leading to L-Lactate and D-Lactate Including Those Not Going Through Lactate Dehydrogenase

**FIGURE 6:**
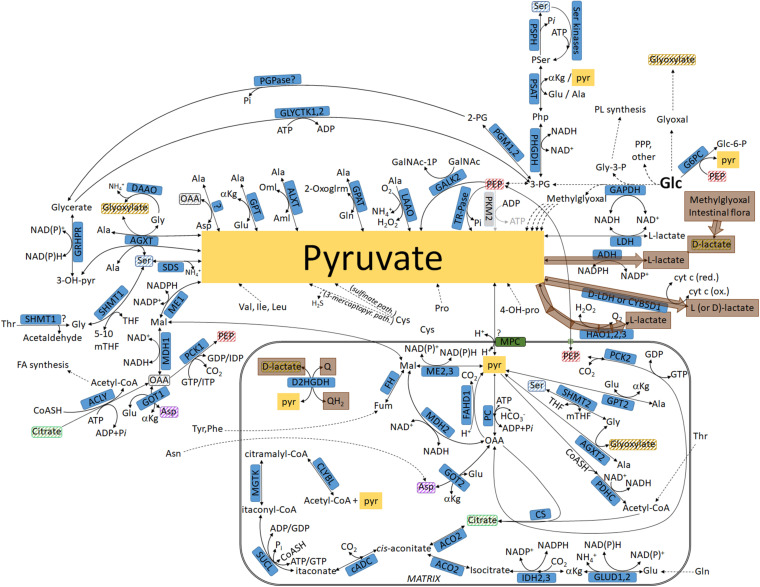
Pathways leading to L- and D-lactate, including those not going through lactate dehydrogenase, highlighted in brown. For abbreviations, see Table 1.

These pathways are shown in Figure 6 (brown arrows).

Lactate—unlike pyruvate—exhibits chirality; thus, it exists in L- or D- configuration. In humans, a putative D-lactate dehydrogenase is known to exist (Flick and Konieczny, 2002; Ewaschuk et al., 2005; Chen et al., 2015). In metabolomics experiments, it is uncommon to distinguish between L- and D-lactate even although it is possible by using special columns. In this section, D- and L-lactate-forming pathways are outlined, including those not going through LDH:

(70) D-lactate formation by methylglyoxal and intestinal flora (Chen et al., 2015) (for considerations related to cancer, see pathway no. 2).

(71) Pyruvate + QH_2_ → D-lactate + Q, catalyzed by D2HGDH in the mitochondrial matrix (Cammack, 1969, 1970). Mutations in D2HGDH have been reported to be involved in multiple types of cancers but render the enzyme hypoactive or inert (Ye et al., 2018); thus, it is unlikely for this route to be important regarding pyruvate production.

(72) D- (or L-) Lactate + 2 ferricytochrome → 2 ferrocytochrome C + 2 H^+^ + pyruvate, catalyzed by D-lactate dehydrogenase; this reaction is mentioned in several databases, but no reference is given.

(73) D- (or L-) Lactate + 2 ferricytochrome → 2 ferrocytochrome C + 2 H^+^ + pyruvate, catalyzed by cytochrome B5 domain-containing protein 1; this reaction is mentioned in several databases, but no reference is given.

(74) Pyruvate + NADPH → NADP^+^ + L-lactate, catalyzed by ADH (Bosron and Prairie, 1972). The many roles of ADH in malignant neoplasms have been extensively reviewed in Orywal and Szmitkowski (2017).

(75) Pyruvate + H_2_O_2_ → L-lactate + O_2_, catalyzed by hydroxyacid oxidases (HAO1,2,3) (Fry and Richardson, 1979; Vignaud et al., 2007). However, in Jones et al. (2000), no HAO activity was reported. In primary pancreatic tumors, HAO3 is strongly downregulated (Thakur et al., 2008). HAO2 was reported to inhibit the malignancy of clear cell renal cell carcinoma cells. Overall, it is unlikely for this to be a substantial pathway in yielding pyruvate in cancer.

(76) Protein deglycase (E.C. 3.5.1.124) may form D-lactate from proteins (Richarme et al., 2015; Richarme and Dairou, 2017). Relevant to this, the deglycase DJ-1/Park7 is important for cancer cell survival (Vasseur et al., 2009).

(77) Methylglyoxal spontaneously forms a hemithioacetal adduct with GSH; subsequently, glyoxalase I (lactoylglutathione lyase; EC 4.4.1.5) produces S-D-lactoylglutathione from this adduct (Thornalley, 1990), and glyoxalase II (hydroxyacylglutathione hydrolase; EC 3.1.2.6), in turn, hydrolyzes S-D-lactoylglutathione to D-lactate + GSH (Cordell et al., 2004) (for considerations related to cancer, see pathway no. 2).

Finally, it is worth mentioning that LDH may process substrates other than pyruvate and lactate, interconverting glyoxylate + NAD^+^ to oxalate + NADH or α-ketobutyrate to →-hydroxybutyrate or L-glycerate to hydroxypyruvate (Dawkins and Dickens, 1965; Kim and Whitesides, 1988).

## Pathways Leading to Pyruvate Commencing From Glutamine (Glutaminolysis)

**FIGURE 7:**
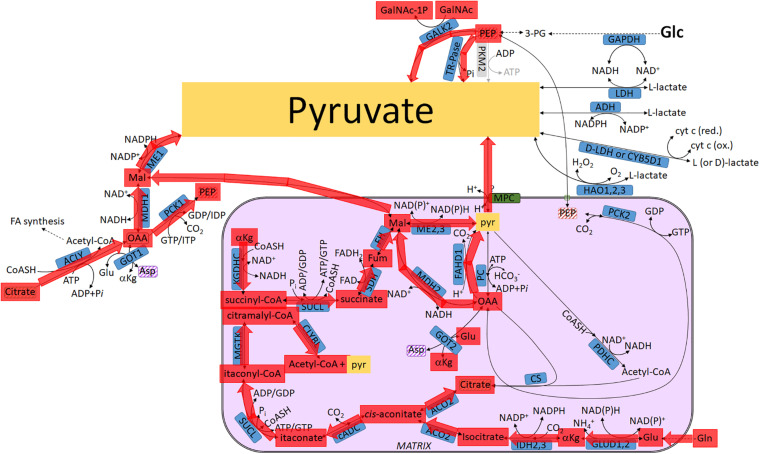
Pathways leading to pyruvate commencing from glutamine (glutaminolysis), highlighted in red. For abbreviations, see Table 1.

It is a well-known fact that most cancer cells grow much better when feeding media contain glutamine; this spurred from the pioneering studies of Eagle et al. (1956), showing the dependence of cancer cells growing in monolayer cultures on glutamine. The many critical roles of glutamine in tumor metabolism is reviewed in Altman et al. (2016). From the energetic point of view it were Reitzer et al. (1979) who first showed that glutamine, not sugars, is the main energy source in cultured HeLa cells and that carbon atoms from glutamine incorporate into lactate, but not more than 13%. Zielke et al. (1980), likewise reported that human diploid fibroblasts metabolize up to 13% of media glutamine to lactate. In the same line of thought, Scott et al. (2011), showed that, in human melanoma cell lines, glutamine did not significantly label lactate, in agreement with the data of Ta and Seyfried (2015) reporting that, in a murine glioblastoma cell line, minimal amounts of lactate derived from glutamine were detected. Le et al. (2012), as well as Son et al. (2013) likewise showed that ^13^C-labeled atoms in glutamine appear in lactate also to a minimal extent. However, in a study published by DeBerardinis et al. (2007), ∼60% of the glutamine metabolized by SF188 cells was claimed to be converted to lactate, although they seemed to combine this percentage with that of alanine production. The pathway of converting glutamine to pyruvate (and lactate), referred to by McKeehan (1982) as “glutaminolysis,” has been considered a hallmark of tumor metabolism; however, this is a misconception: in normal tissues, ∼18% of glutamine carbons appear in lactate (Windmueller and Spaeth, 1974), as opposed to ∼10–13% (or less) in tumor cells (see the references above). Thus, if anything, cancer cells exhibit a *decrease* in glutamine-to-lactate conversion exactly as anticipated, mindful that glutamine provides both energy and building blocks for several biosynthetic processes of cancer. Although glutaminolysis was originally attributed to the pathway Gln → Glu → aKg → succinyl-CoA → succinate → fumarate → malate (exiting the mitochondria) → pyruvate (through malic enzyme), several other routes may also contribute (outlined below; see Figure 7).

(78) (For the sake of completion, the glutaminolysis pathway proposed by McKeehan (1982) is repeated in the present entry) Gln → Glu → aKg → succinyl-CoA → succinate → fumarate → malate; malate exits the mitochondria → pyruvate; this last step is catalyzed by cytosolic malic enzyme (ME1).

(79) Gln → Glu → aKg → isocitrate → cis-aconitate → itaconate by cADC; itaconate + CoASH + ATP (or GTP) → itaconyl-CoA + Pi + ADP (or GDP) by SUCL (Nemeth et al., 2016); itaconyl-CoA → citramalyl-CoA by methylglutaconase (MGTK); citramalyl-coA → acetyl-CoA + pyruvate by CLYBL (Shen et al., 2017). Pyruvate may exit the mitochondria through the MPC.

(80) Gln → Glu → aKg → isocitrate → cis-aconitate → citrate, exiting the mitochondria → citrate + ATP + CoASH → acetyl-coA + ADP + Pi + OAA by ACLY (Chypre et al., 2012); OAA → Mal by MDH1; Mal → pyruvate by ME1.

(81) Gln → Glu → aKg → isocitrate → cis-aconitate → citrate, exiting the mitochondria → citrate + ATP + CoASH → acetyl-coA + ADP + Pi + OAA by ACLY; OAA → PEP by PCK1; PEP + GalNAc → GalNAc-1P + pyruvate. The terminal reaction is catalyzed by N-acetylgalactosamine kinase isoforms 1 or 2.

(82) Gln → Glu → aKg → isocitrate → cis-aconitate → citrate, exiting the mitochondria → citrate + ATP + CoASH → acetyl-coA + ADP + Pi + OAA by ACLY; OAA → PEP by PCK1; PEP → pyruvate; the terminal reaction is catalyzed by tartrate-resistant acid phosphatases.

(83) Gln → Glu → aKg → succinyl-CoA → succinate → fumarate → malate → pyruvate by ME2,3; pyruvate may exit the mitochondria through the MPC.

(84) Gln → Glu → aKg; aKg transaminates with Asp forming Glu and OAA, by GOT2; OAA → pyruvate by FAHD1 (Pircher et al., 2011, 2015); pyruvate may exit the mitochondria through the MPC.

(85) Gln → Glu → aKg; aKg transaminates with Asp forming Glu and OAA, by GOT2; OAA → Mal by MDH2; Mal exits the mitochondria; Mal → pyruvate by ME1 (Zelewski and Swierczynski, 1991; Loeber et al., 1994).

(86) Gln → Glu → aKg; aKg transaminates with Asp forming Glu and OAA, by GOT2; OAA → Mal by MDH2; malate → pyruvate by ME2,3; pyruvate may exit the mitochondria through the MPC.

## Energetics of Glycolysis With Kinetically Inactive PK

Glycolysis yields a net of two ATP molecules per glucose molecule; however, in view of an inactive PK while pyruvate is made through PK-bypass pathways, net ATP production from glycolysis is expected to be zero. Although the importance of high-energy phosphate generation has been downplayed in cancer tissues (Vander Heiden et al., 2009), it cannot be ignored that—according to the BRENDA database—among the 336 enzymatic reactions requiring ATP in a cell (without even considering quantitatively important, non-enzymatic mechanisms such as Na^+^/K^+^ ATPase), 125 of them occur in the cytosol. Clearly, while it is imperative to prevent phosphofructokinase and hexokinase from ATP-dependent feedback inhibition and allow a high flux of glycolysis for the sake of generating intermediates shuttled toward other pathways, ATP is still needed for many other reactions. Crunching the numbers regarding cytosolic energetics is a daunting task, but what is definite is that a cell with nearly zero ATP production from glycolysis may not harbor ATP-consuming mitochondria, for whatever reason (hypoxia, mtDNA mutations, etc.). This can be solved by maintaining the adenine nucleotide translocase in “forward” mode, i.e., providing ATP to the cytosol which is made by SUCL supported by glutaminolysis (Chinopoulos et al., 2010). Production of pyruvate and, therefore lactate is still maintained by the PK-bypassing pathways so as to thwart a reductive stress as pyruvate-to-lactate by LDH maintains a low NADH/NAD^+^ ratio. Finally, it is important to emphasize that this lack of ATP generation by glycolysis due to PK inhibition does not only occur in neoplastic tissues, but it seems to be a more general pathophysiological mechanism also present in tissue ischemia: it was recently reported that during acute kidney injury, PK was inhibited by oxidative/nitrosative stress for the purpose of diverting glycolytic intermediates toward the pentose phosphate pathway which, in turn, yielded reducing equivalents and mounted a better response during the reperfusion phase where ROS are formed, thus increasing the chances for organ survival (Zhou et al., 2019).

## Conclusion

The above considerations aim to (i) highlight that L-lactate can still be produced from pyruvate using carbon atoms originating from glucose or other substrates in cells with kinetically impaired pyruvate kinase and (ii) show that the mitochondria may contribute to cancer metabolism irrespective of oxidative phosphorylation by providing means of contributing to pyruvate production. Having said that, it is important to emphasize that none of the aforementioned reactions take into account the potential regulatory effects of metabolites on other reactions such as those occurring on PK by amino acids (Chaneton et al., 2012; Yuan et al., 2018). In addition, each enzyme probably exhibits different kinetic and thermodynamic constraints which control the overall flux, which also means that many of these pathways may not operate simultaneously. Such exponentially increasing complexity of a system precludes the possibility of predictions and modeling, though I would be happy to be proven wrong.

## Author Contributions

CC wrote and edited the manuscript.

## Conflict of Interest

The author declares that the research was conducted in the absence of any commercial or financial relationships that could be construed as a potential conflict of interest.

## Footnotes

1 https://www.genome.jp/kegg/

2 www.brenda-enzymes.org

3 https://www.metabolicatlas.org/

4 http://bigg.ucsd.edu/

5 http://www.metanetx.org/

6 www.hmdb.ca

7 https://www.vmh.life/

8 https://cancer.sanger.ac.uk/cosmic/gene/analysis?ln=CLYBL

## References

[B1] Abbadi S. Rodarte J. J. Abutaleb A. Lavell E. Smith C. L. Ruff W. (2014). Glucose-6-phosphatase is a key metabolic regulator of glioblastoma invasion. *Mol. Cancer Res.* 12 1547–1559. 10.1158/1541-7786.mcr-14-0106-t 25001192 PMC4233174

[B2] Abe H. Yoshikawa N. Sarower M. G. Okada S. (2005). Physiological function and metabolism of free D-alanine in aquatic animals. *Biol. Pharm. Bull.* 28 1571–1577. 10.1248/bpb.28.1571 16141518

[B3] Alquraishi M. Puckett D. L. Alani D. S. Humidat A. S. Frankel V. D. Donohoe D. R. (2019). Pyruvate kinase M2: a simple molecule with complex functions. *Free Radic. Biol. Med.* 143 176–192. 10.1016/j.freeradbiomed.2019.08.007 31401304 PMC6848794

[B4] Altenberg B. Greulich K. O. (2004). Genes of glycolysis are ubiquitously overexpressed in 24 cancer classes. *Genomics* 84 1014–1020. 10.1016/j.ygeno.2004.08.010 15533718

[B5] Altman B. J. Stine Z. E. Dang C. V. (2016). From Krebs to clinic: glutamine metabolism to cancer therapy. *Nat. Rev. Cancer* 16 619–634. 10.1038/nrc.2016.71 27492215 PMC5484415

[B6] Ananieva E. A. Wilkinson A. C. (2018). Branched-chain amino acid metabolism in cancer. *Curr. Opin. Clin. Nutr. Metab. Care* 21 64–70. 10.1097/mco.0000000000000430 29211698 PMC5732628

[B7] Anastasiou D. Poulogiannis G. Asara J. M. Boxer M. B. Jiang J. K. Shen M. (2011). Inhibition of pyruvate kinase M2 by reactive oxygen species contributes to cellular antioxidant responses. *Science* 334 1278–1283. 10.1126/science.1211485 22052977 PMC3471535

[B8] Anastasiou D. Yu Y. Israelsen W. J. Jiang J. K. Boxer M. B. Hong B. S. (2012). Pyruvate kinase M2 activators promote tetramer formation and suppress tumorigenesis. *Nat. Chem. Biol.* 8 839–847. 22922757 10.1038/nchembio.1060 PMC3711671

[B9] Antognelli C. Moretti S. Frosini R. Puxeddu E. Sidoni A. Talesa V. N. (2019). Methylglyoxal Acts as a Tumor-Promoting Factor in Anaplastic Thyroid Cancer. *Cells* 8 547. 10.3390/cells8060547 31174324 PMC6627963

[B10] Baker P. R. Cramer S. D. Kennedy M. Assimos D. G. Holmes R. P. (2004). Glycolate and glyoxylate metabolism in HepG2 cells. *Am. J. Physiol. Cell Physiol.* 287 C1359–C1365. 15240345 10.1152/ajpcell.00238.2004

[B11] Baranowski T. Wolna E. Morawiecki A. (1968). Purification and properties of crystalline 2-phospho-D-glycerate hydro-lyase from human muscle. *Eur. J. Biochem.* 5 119–123. 10.1111/j.1432-1033.1968.tb00345.x 5660677

[B12] Bartesaghi S. Graziano V. Galavotti S. Henriquez N. V. Betts J. Saxena J. (2015). Inhibition of oxidative metabolism leads to p53 genetic inactivation and transformation in neural stem cells. *Proc. Natl. Acad. Sci. U.S.A.* 112 1059–1064. 10.1073/pnas.1413165112 25583481 PMC4313844

[B13] Bastings J. van Eijk H. M. Olde Damink S. W. Rensen S. S. (2019). d-amino acids in health and disease: a focus on cancer. *Nutrients* 11:2205. 10.3390/nu11092205 31547425 PMC6770864

[B14] Bensard C. L. Wisidagama D. R. Olson K. A. Berg J. A. Krah N. M. Schell J. C. (2020). Regulation of tumor initiation by the mitochondrial Pyruvate Carrier. *Cell Metab.* 31 284–300.e7. 31813825 10.1016/j.cmet.2019.11.002 PMC7004878

[B15] Bhandari R. K. Oda R. P. Petrikovics I. Thompson D. E. Brenner M. Mahon S. B. (2014). Cyanide toxicokinetics: the behavior of cyanide, thiocyanate and 2-amino-2-thiazoline-4-carboxylic acid in multiple animal models. *J. Anal. Toxicol.* 38 218–225. 10.1093/jat/bku020 24711295 PMC3977587

[B16] Bose S. Le A. (2018). Glucose metabolism in cancer. *Adv. Exp. Med. Biol.* 1063 3–12. 29946772 10.1007/978-3-319-77736-8_1

[B17] Bosron W. F. Prairie R. L. (1972). Triphosphopyridine nucleotide-linked aldehyde reductase. I. Purification and properties of the enzyme from pig kidney cortex. *J. Biol. Chem.* 247 4480–4485. 4402936

[B18] Brunetti P. Jourdian G. W. Roseman S. (1962). The sialic acids. III. Distribution and properties of animal N-acetylneuraminic aldolase. *J. Biol. Chem.* 237 2447–2453. 13874013

[B19] Bukato G. Kochan Z. Swierczynski J. (1995). Purification and properties of cytosolic and mitochondrial malic enzyme isolated from human brain. *Int. J. Biochem. Cell Biol.* 27 47–54. 10.1016/1357-2725(94)00057-3 7757881

[B20] Bull H. Murray P. G. Thomas D. Fraser A. M. Nelson P. N. (2002). Acid phosphatases. *Mol. Pathol.* 55 65–72. 11950951 10.1136/mp.55.2.65 PMC1187150

[B21] Cammack R. (1969). Assay, purification and properties of mammalian D-2-hydroxy acid dehydrogenase. *Biochem. J.* 115 55–64. 10.1042/bj1150055 5359443 PMC1185068

[B22] Cammack R. (1970). Mammalian D-2-hydroxy acid dehydrogenase. Effect of inhibitors and reaction sequence. *Biochem. J.* 118 405–408. 10.1042/bj1180405 5528639 PMC1179206

[B23] Cao S. Durrani F. A. Toth K. Rustum Y. M. (2014). Se-methylselenocysteine offers selective protection against toxicity and potentiates the antitumour activity of anticancer drugs in preclinical animal models. *Br. J. Cancer* 110 1733–1743. 10.1038/bjc.2014.85 24619073 PMC3974093

[B24] Capala M. E. Pruis M. Vellenga E. Schuringa J. J. (2016). Depletion of SAM50 Specifically Targets BCR-ABL-expressing leukemic stem and progenitor cells by interfering with mitochondrial functions. *Stem Cells Dev.* 25 427–437. 10.1089/scd.2015.0151 26855047

[B25] Carlsen B. D. Lambeth D. O. Ray P. D. (1988). Synthesis of malate from phosphoenolpyruvate by rabbit liver mitochondria: implications for lipogenesis. *Biochim. Biophys. Acta* 965 1–8. 10.1016/0304-4165(88)90143-2 2831992

[B26] Carlson G. M. Colombo G. Lardy H. A. (1978). A vicinal dithiol containing an essential cysteine in phosphoenolpyruvate carboxykinase (guanosine triphosphate) from cytosol of rat liver. *Biochemistry* 17 5329–5338. 10.1021/bi00618a002 728403

[B27] Castellano F. Molinier-Frenkel V. (2017). An overview of l-amino acid oxidase functions from bacteria to mammals: focus on the immunoregulatory phenylalanine oxidase IL4I1. *Molecules* 22:2151. 10.3390/molecules22122151 29206151 PMC6149928

[B28] Chaneton B. Hillmann P. Zheng L. Martin A. C. L. Maddocks O. D. K. Chokkathukalam A. (2012). Serine is a natural ligand and allosteric activator of pyruvate kinase M2. *Nature* 491 458–462. 10.1038/nature11540 23064226 PMC3894725

[B29] Chao T. Y. Yu J. C. Ku C. H. Chen M. M. Lee S. H. Janckila A. J. (2005). Tartrate-resistant acid phosphatase 5b is a useful serum marker for extensive bone metastasis in breast cancer patients. *Clin. Cancer Res.* 11 544–550. 15701839

[B30] Chen C. H. Chen S. C. (1988). Evidence of acid phosphatase in the cytoplasm as a distinct entity. *Arch. Biochem. Biophys.* 262 427–438. 10.1016/0003-9861(88)90394-3 3364974

[B31] Chen C. M. Chen S. M. Chien P. J. Yu H. Y. (2015). Development of an enzymatic assay system of D-lactate using D-lactate dehydrogenase and a UV-LED fluorescent spectrometer. *J. Pharm. Biomed. Anal.* 116 150–155. 10.1016/j.jpba.2015.07.018 26265307

[B32] Chiavarina B. Nokin M. J. Bellier J. Durieux F. Bletard N. Sherer F. (2017). Methylglyoxal-mediated stress correlates with high metabolic activity and promotes tumor growth in colorectal cancer. *Int. J. Mol. Sci.* 18:213. 10.3390/ijms18010213 28117708 PMC5297842

[B33] Chiku T. Padovani D. Zhu W. Singh S. Vitvitsky V. Banerjee R. (2009). H2S biogenesis by human cystathionine gamma-lyase leads to the novel sulfur metabolites lanthionine and homolanthionine and is responsive to the grade of hyperhomocysteinemia. *J. Biol. Chem.* 284 11601–11612. 10.1074/jbc.m808026200 19261609 PMC2670165

[B34] Chinopoulos C. Gerencser A. A. Mandi M. Mathe K. Torocsik B. Doczi J. (2010). Forward operation of adenine nucleotide translocase during F0F1-ATPase reversal: critical role of matrix substrate-level phosphorylation. *FASEB J.* 24 2405–2416. 10.1096/fj.09-149898 20207940 PMC2887268

[B35] Christofk H. R. Vander Heiden M. G. Harris M. H. Ramanathan A. Gerszten R. E. Wei R. (2008). The M2 splice isoform of pyruvate kinase is important for cancer metabolism and tumour growth. *Nature* 452 230–233. 10.1038/nature06734 18337823

[B36] Chypre M. Zaidi N. Smans K. (2012). ATP-citrate lyase: a mini-review. *Biochem. Biophys. Res. Commun.* 422 1–4. 10.1016/j.bbrc.2012.04.144 22575446

[B37] Colilla W. Jorgenson R. A. Nordlie R. C. (1975). Mammalian carbamyl phosphate : glucose phosphotransferase and glucose-6-phosphate phosphohydrolase: extended tissue distribution. *Biochim. Biophys. Acta* 377 117–125. 10.1016/0005-2744(75)90292-2 164220

[B38] Collins M. A. Nijm W. P. Borge G. F. Teas G. Goldfarb C. (1979). Dopamine-related tetrahydroisoquinolines: significant urinary excretion by alcoholics after alcohol consumption. *Science* 206 1184–1186. 10.1126/science.505002 505002

[B39] Cooper A. J. (1977). Asparagine transaminase from rat liver. *J. Biol. Chem.* 252 2032–2038. 14957

[B40] Cooper A. J. Krasnikov B. F. Niatsetskaya Z. V. Pinto J. T. Callery P. S. Villar M. T. (2011). Cysteine S-conjugate beta-lyases: important roles in the metabolism of naturally occurring sulfur and selenium-containing compounds, xenobiotics and anticancer agents. *Amino Acids* 41 7–27. 10.1007/s00726-010-0552-0 20306345 PMC2898922

[B41] Cooper A. J. Kuhara T. (2014). alpha-Ketoglutaramate: an overlooked metabolite of glutamine and a biomarker for hepatic encephalopathy and inborn errors of the urea cycle. *Metab. Brain Dis.* 29 991–1006. 10.1007/s11011-013-9444-9 24234505 PMC4020999

[B42] Cooper A. J. Pinto J. T. (2006). Cysteine S-conjugate beta-lyases. *Amino Acids* 30 1–15. 10.1007/s00726-005-0243-4 16463021

[B43] Cooper A. J. Raps S. P. Meister A. (1987). Fluorometric determination of alpha-ketosuccinamic acid in rat tissues. *Anal. Biochem.* 167 312–320. 10.1016/0003-2697(87)90170-9 3442326

[B44] Cooper J. L. Meister A. (1972). Isolation and properties of highly purified glutamine transaminase. *Biochemistry* 11 661–671. 10.1021/bi00755a001 5059882

[B45] Copper A. J. Meister A. (1973). Enzymatic conversion of O-carbamyl-L-serine to pyruvate and ammonia. *Biochem. Biophys. Res. Commun.* 55 780–787. 10.1016/0006-291x(73)91212-6 4761084

[B46] Cordell P. A. Futers T. S. Grant P. J. Pease R. J. (2004). The Human hydroxyacylglutathione hydrolase (HAGH) gene encodes both cytosolic and mitochondrial forms of glyoxalase II. *J. Biol. Chem.* 279 28653–28661. 10.1074/jbc.m403470200 15117945

[B47] Cortes-Cros M. Hemmerlin C. Ferretti S. Zhang J. Gounarides J. S. Yin H. (2013). M2 isoform of pyruvate kinase is dispensable for tumor maintenance and growth. *Proc. Natl. Acad. Sci. U.S.A.* 110 489–494. 10.1073/pnas.1212780110 23267074 PMC3545759

[B48] D’Aniello C. Patriarca E. J. Phang J. M. Minchiotti G. (2020). Proline metabolism in tumor growth and metastatic progression. *Front. Oncol.* 10:776. 10.3389/fonc.2020.00776 32500033 PMC7243120

[B49] Danpure C. J. Lumb M. J. Birdsey G. M. Zhang X. (2003). Alanine:glyoxylate aminotransferase peroxisome-to-mitochondrion mistargeting in human hereditary kidney stone disease. *Biochim. Biophys. Acta* 1647 70–75. 10.1016/s1570-9639(03)00055-4 12686111

[B50] Dawkins P. D. Dickens F. (1965). The Oxidation of D- and L-Glycerate by Rat Liver. *Biochem. J.* 94 353–367. 10.1042/bj0940353 14346088 PMC1206517

[B51] Dayton T. L. Gocheva V. Miller K. M. Bhutkar A. Lewis C. A. Bronson R. T. (2018). Isoform-specific deletion of PKM2 constrains tumor initiation in a mouse model of soft tissue sarcoma. *Cancer Metab.* 6:6. 10.1186/s40170-018-0179-2 PMC5977456 29854399

[B52] Dayton T. L. Gocheva V. Miller K. M. Israelsen W. J. Bhutkar A. Clish C. B. (2016a). Germline loss of PKM2 promotes metabolic distress and hepatocellular carcinoma. *Genes Dev.* 30 1020–1033. 10.1101/gad.278549.116 27125672 PMC4863734

[B53] Dayton T. L. Jacks T. Vander Heiden M. G. (2016b). PKM2, cancer metabolism, and the road ahead. *EMBO Rep.* 17 1721–1730. 27856534 10.15252/embr.201643300 PMC5283597

[B54] de la Cruz-Lopez K. G. Castro-Munoz L. J. Reyes-Hernandez D. O. Garcia-Carranca A. Manzo-Merino J. (2019). Lactate in the regulation of tumor microenvironment and therapeutic approaches. *Front. Oncol.* 9:1143. 10.3389/fonc.2019.01143 31737570 PMC6839026

[B55] De Marchi T. Timmermans M. A. Sieuwerts A. M. Smid M. Look M. P. Grebenchtchikov N. (2017). Phosphoserine aminotransferase 1 is associated to poor outcome on tamoxifen therapy in recurrent breast cancer. *Sci. Rep.* 7:2099. 10.1038/s41598-017-02296-w PMC5437008 28522855

[B56] DeBerardinis R. J. Lum J. J. Hatzivassiliou G. Thompson C. B. (2008). The biology of cancer: metabolic reprogramming fuels cell growth and proliferation. *Cell Metab.* 7 11–20. 10.1016/j.cmet.2007.10.002 18177721

[B57] DeBerardinis R. J. Mancuso A. Daikhin E. Nissim I. Yudkoff M. Wehrli S. (2007). Beyond aerobic glycolysis: transformed cells can engage in glutamine metabolism that exceeds the requirement for protein and nucleotide synthesis. *Proc. Natl. Acad. Sci. U.S.A.* 104 19345–19350. 10.1073/pnas.0709747104 18032601 PMC2148292

[B58] Dey P. Baddour J. Muller F. Wu C. C. Wang H. Liao W. T. (2017). Genomic deletion of malic enzyme 2 confers collateral lethality in pancreatic cancer. *Nature* 542 119–123. 10.1038/nature21052 28099419 PMC5398413

[B59] Dong Y. Tu R. Liu H. Qing G. (2020). Regulation of cancer cell metabolism: oncogenic MYC in the driver’s seat. *Signal Transduct. Target. Ther.* 5:124. 10.1038/s41392-020-00235-2 PMC7351732 32651356

[B60] Drahota Z. Rauchova H. Mikova M. Kaul P. Bass A. (1983). Phosphoenolpyruvate shuttle–transport of energy from mitochondria to cytosol. *FEBS Lett.* 157 347–349. 10.1016/0014-5793(83)80573-0 6862029

[B61] Ducker G. S. Ghergurovich J. M. Mainolfi N. Suri V. Jeong S. K. Hsin-Jung Li S. (2017). Human SHMT inhibitors reveal defective glycine import as a targetable metabolic vulnerability of diffuse large B-cell lymphoma. *Proc. Natl. Acad. Sci. U.S.A.* 114 11404–11409. 10.1073/pnas.1706617114 29073064 PMC5664509

[B62] Eagle H. Oyama V. I. Levy M. Horton C. L. Fleischman R. (1956). The growth response of mammalian cells in tissue culture to L-glutamine and L-glutamic acid. *J. Biol. Chem.* 218 607–616. 13295214

[B63] Elia I. Broekaert D. Christen S. Boon R. Radaelli E. Orth M. F. (2017). Proline metabolism supports metastasis formation and could be inhibited to selectively target metastasizing cancer cells. *Nat. Commun.* 8:15267. 10.1038/ncomms15267 PMC5437289 28492237

[B64] Erecinska M. Wilson D. F. (1984). Relationship of the intra- and extramitochondrial adenine nucleotide ratios during synthesis of phosphoenolpyruvate using extramitochondrial ATP. *J. Biol. Chem.* 259 10904–10906. 6088521

[B65] Esaki N. Nakamura T. Tanaka H. Soda K. (1982). Selenocysteine lyase, a novel enzyme that specifically acts on selenocysteine. Mammalian distribution and purification and properties of pig liver enzyme. *J. Biol. Chem.* 257 4386–4391. 6461656

[B66] Ewaschuk J. B. Naylor J. M. Zello G. A. (2005). D-lactate in human and ruminant metabolism. *J. Nutr.* 135 1619–1625. 10.1093/jn/135.7.1619 15987839

[B67] Feng H. Wang X. Chen J. Cui J. Gao T. Gao Y. (2019). Nuclear imaging of glucose metabolism: beyond (18)F-FDG. *Contrast Media Mol. Imaging* 2019:7954854. 10.1155/2019/7954854 PMC6458935 31049045

[B68] Finak G. Bertos N. Pepin F. Sadekova S. Souleimanova M. Zhao H. (2008). Stromal gene expression predicts clinical outcome in breast cancer. *Nat. Med.* 14 518–527. 18438415 10.1038/nm1764

[B69] Flick M. J. Konieczny S. F. (2002). Identification of putative mammalian D-lactate dehydrogenase enzymes. *Biochem. Biophys. Res. Commun.* 295 910–916. 10.1016/s0006-291x(02)00768-4 12127981

[B70] Fong M. Y. McDunn J. Kakar S. S. (2011). Identification of metabolites in the normal ovary and their transformation in primary and metastatic ovarian cancer. *PLoS One* 6:e19963. 10.1371/journal.pone.0019963 21625518 PMC3098284

[B71] Fry D. W. Richardson K. E. (1979). Isolation and characterization of glycolic acid oxidase from human liver. *Biochim. Biophys. Acta* 568 135–144. 10.1016/0005-2744(79)90281-x 444540

[B72] Fuchs S. A. Berger R. Klomp L. W. de Koning T. J. (2005). D-amino acids in the central nervous system in health and disease. *Mol. Genet. Metab.* 85 168–180. 10.1016/j.ymgme.2005.03.003 15979028

[B73] Garber A. J. Ballard F. J. (1970). Regulation of phosphoenolpyruvate metabolism in mitochondria from guinea pig liver. *J. Biol. Chem.* 245 2229–2240. 4315147

[B74] Garber A. J. Salganicoff L. (1973). Regulation of oxalacetate metabolism in liver mitochondria. Evidence for nicotinamide adenine dinucleotide-malate dehydrogenase equilibrium and the role of phosphoenolpyruvate carboxykinase in the control of oxalacetate metabolism in intact guinea pig and rat liver mitochondria. *J. Biol. Chem.* 248 1520–1529. 4144388

[B75] Garrow T. A. Brenner A. A. Whitehead V. M. Chen X. N. Duncan R. G. Korenberg J. R. (1993). Cloning of human cDNAs encoding mitochondrial and cytosolic serine hydroxymethyltransferases and chromosomal localization. *J. Biol. Chem.* 268 11910–11916. 8505317

[B76] Gavaret J. M. Nunez J. Cahnmann H. J. (1980). Formation of dehydroalanine residues during thyroid hormone synthesis in thyroglobulin. *J. Biol. Chem.* 255 5281–5285. 7372636

[B77] Goto Y. Shimizu J. Shukuya R. (1980). Purification and molecular characteristics of mitochondrial phosphoenolpyruvate carboxykinase from bullfrog (*Rana catesbeiana*) liver. *J. Biochem.* 88 1239–1249. 10.1093/oxfordjournals.jbchem.a133092 6970195

[B78] Guo J. H. Hexige S. Chen L. Zhou G. J. Wang X. Jiang J. M. (2006). Isolation and characterization of the human D-glyceric acidemia related glycerate kinase gene GLYCTK1 and its alternatively splicing variant GLYCTK2. *DNA Seq.* 17 1–7. 10.1080/10425170500476665 16753811

[B79] Hall A. Meyle K. D. Lange M. K. Klima M. Sanderhoff M. Dahl C. (2013). Dysfunctional oxidative phosphorylation makes malignant melanoma cells addicted to glycolysis driven by the (V600E)BRAF Oncogene. *Oncotarget* 4 584–599. 10.18632/oncotarget.965 23603840 PMC3720606

[B80] Harris R. A. Fenton A. W. (2019). A critical review of the role of M2PYK in the Warburg effect. *Biochim. Biophys. Acta Rev. Cancer* 1871 225–239. 10.1016/j.bbcan.2019.01.004 30708038 PMC6525063

[B81] Hayman A. R. Warburton M. J. Pringle J. A. Coles B. Chambers T. J. (1989). Purification and characterization of a tartrate-resistant acid phosphatase from human osteoclastomas. *Biochem. J.* 261 601–609. 10.1042/bj2610601 2775236 PMC1138867

[B82] Hebda C. A. Nowak T. (1982). The purification, characterization, and activation of phosphoenolpyruvate carboxykinase from chicken liver mitochondria. *J. Biol. Chem.* 257 5503–5514. 7068603

[B83] Helwig J. J. Farooqui A. A. Bollack C. Mandel P. (1978). Purification and some properties of tartrate-sensitive acid phosphatase from rabbit kidney cortex. *Biochem. J.* 175 321–329. 10.1042/bj1750321 736900 PMC1186068

[B84] Hillis A. L. Lau A. N. Devoe C. X. Dayton T. L. Danai L. V. Di Vizio D. (2018). PKM2 is not required for pancreatic ductal adenocarcinoma. *Cancer Metab.* 6:17. 10.1186/s40170-018-0188-1 PMC6198443 30386596

[B85] Hirsch H. Greenberg D. M. (1967). Studies on phosphoserine aminotransferase of sheep brain. *J. Biol. Chem.* 242 2283–2287. 6022873

[B86] Holmes R. P. Assimos D. G. (1998). Glyoxylate synthesis, and its modulation and influence on oxalate synthesis. *J. Urol.* 160 1617–1624. 10.1097/00005392-199811000-00003 9783918

[B87] Hong C. S. Graham N. A. Gu W. Espindola Camacho C. Mah V. Maresh E. L. (2016). MCT1 Modulates Cancer Cell Pyruvate Export and Growth of Tumors that Co-express MCT1 and MCT4. *Cell Rep.* 14 1590–1601. 10.1016/j.celrep.2016.01.057 26876179 PMC4816454

[B88] Hoshino A. Hirst J. A. Fujii H. (2007). Regulation of cell proliferation by interleu-induced nuclear translocation of pyruvate kinase. *J. Biol. Chem.* 282 17706–17711. 10.1074/jbc.m700094200 17446165

[B89] Hosios A. M. Fiske B. P. Gui D. Y. Vander Heiden M. G. (2015). Lack of Evidence for PKM2 Protein Kinase Activity. *Mol. Cell* 59 850–857. 10.1016/j.molcel.2015.07.013 26300261 PMC4548833

[B90] Hsu M. C. Hung W. C. (2018). Pyruvate kinase M2 fuels multiple aspects of cancer cells: from cellular metabolism, transcriptional regulation to extracellular signaling. *Mol. Cancer* 17:35. 10.1186/s12943-018-0791-3 PMC5817853 29455645

[B91] Hu Y. Lu W. Chen G. Wang P. Chen Z. Zhou Y. (2012). K-ras(G12V) transformation leads to mitochondrial dysfunction and a metabolic switch from oxidative phosphorylation to glycolysis. *Cell Res.* 22 399–412. 10.1038/cr.2011.145 21876558 PMC3257361

[B92] Hussien R. Brooks G. A. (2011). Mitochondrial and plasma membrane lactate transporter and lactate dehydrogenase isoform expression in breast cancer cell lines. *Physiol. Genomics* 43 255–264. 10.1152/physiolgenomics.00177.2010 21177384 PMC3068517

[B93] Icard P. Lincet H. (2012). A global view of the biochemical pathways involved in the regulation of the metabolism of cancer cells. *Biochim. Biophys. Acta* 1826 423–433. 10.1016/j.bbcan.2012.07.001 22841746

[B94] Ip C. Hayes C. Budnick R. M. Ganther H. E. (1991). Chemical form of selenium, critical metabolites, and cancer prevention. *Cancer Res.* 51 595–600. 1824684

[B95] Israelsen W. J. Dayton T. L. Davidson S. M. Fiske B. P. Hosios A. M. Bellinger G. (2013). PKM2 isoform-specific deletion reveals a differential requirement for pyruvate kinase in tumor cells. *Cell* 155 397–409. 10.1016/j.cell.2013.09.025 24120138 PMC3850755

[B96] Israelsen W. J. Vander Heiden M. G. (2015). Pyruvate kinase: function, regulation and role in cancer. *Semin. Cell Dev. Biol.* 43 43–51. 10.1016/j.semcdb.2015.08.004 26277545 PMC4662905

[B97] Ito S. Ohyama T. Kontani Y. Matslida K. Sakata S. F. Tamaki N. (2001). Influence of dietary protein levels on beta-alanine aminotransferase expression and activity in rats. *J. Nutr. Sci. Vitaminol.* 47 275–282. 10.3177/jnsv.47.275 11767207

[B98] Jeske L. Placzek S. Schomburg I. Chang A. Schomburg D. (2019). BRENDA in 2019: a European ELIXIR core data resource. *Nucleic Acids Res.* 47 D542–D549. 30395242 10.1093/nar/gky1048 PMC6323942

[B99] Jones J. M. Morrell J. C. Gould S. J. (2000). Identification and characterization of HAOX1, HAOX2, and HAOX3, three human peroxisomal 2-hydroxy acid oxidases. *J. Biol. Chem.* 275 12590–12597. 10.1074/jbc.275.17.12590 10777549

[B100] Kakimoto Y. Taniguchi K. Sano I. (1969). D-beta-aminoisobutyrate:pyruvate aminotransferase in mammalian liver and excretion of beta-aminoisobutyrate by man. *J. Biol. Chem.* 244 335–340. 5773299

[B101] Kanehisa M. Goto S. (2000). KEGG: kyoto encyclopedia of genes and genomes. *Nucleic Acids Res.* 28 27–30. 10592173 10.1093/nar/28.1.27 PMC102409

[B102] Kashii T. Gomi T. Oya T. Ishii Y. Oda H. Maruyama M. (2005). Some biochemical and histochemical properties of human liver serine dehydratase. *Int. J. Biochem. Cell Biol.* 37 574–589. 10.1016/j.biocel.2004.08.004 15618015

[B103] Kim M. J. Whitesides G. M. (1988). L-Lactate dehydrogenase: substrate specificity and use as a catalyst in the synthesis of homochiral 2-hydroxy acids. *J. Am. Chem. Soc.* 110 2959–2964. 10.1021/ja00217a044 2610514

[B104] King Z. A. Lu J. Drager A. Miller P. Federowicz S. Lerman J. A. (2016). BiGG Models: a platform for integrating, standardizing and sharing genome-scale models. *Nucleic Acids Res.* 44 D515–D522. 26476456 10.1093/nar/gkv1049 PMC4702785

[B105] Kleineke J. Sauer H. Soling H. D. (1973). On the specificity of the tricarboxylate carrier system in rat liver mitochondria. *FEBS Lett.* 29 82–86. 10.1016/0014-5793(73)80531-9 4719206

[B106] Knott S. R. V. Wagenblast E. Khan S. Kim S. Y. Soto M. Wagner M. (2018). Asparagine bioavailability governs metastasis in a model of breast cancer. *Nature* 554 378–381. 10.1038/nature25465 29414946 PMC5898613

[B107] Koizumi M. Ogata E. (2002). Bone metabolic markers as gauges of metastasis to bone: a review. *Ann. Nucl. Med.* 16 161–168. 10.1007/bf02996296 12126040

[B108] Koseki J. Konno M. Asai A. Colvin H. Kawamoto K. Nishida N. (2018). Enzymes of the one-carbon folate metabolism as anticancer targets predicted by survival rate analysis. *Sci. Rep.* 8:303. 10.1038/s41598-017-18456-x PMC5762868 29321536

[B109] Krall A. S. Xu S. Graeber T. G. Braas D. Christofk H. R. (2016). Asparagine promotes cancer cell proliferation through use as an amino acid exchange factor. *Nat. Commun.* 7:11457. 10.1038/ncomms11457 PMC4855534 27126896

[B110] Krebs H. A. Johnson W. A. (1937). Acetopyruvic acid (alphagamma-diketovaleric acid) as an intermediate metabolite in animal tissues. *Biochem. J.* 31 772–779. 10.1042/bj0310772 16746397 PMC1267003

[B111] Lambrecht J. A. Flynn J. M. Downs D. M. (2012). Conserved YjgF protein family deaminates reactive enamine/imine intermediates of pyridoxal 5’-phosphate (PLP)-dependent enzyme reactions. *J. Biol. Chem.* 287 3454–3461. 10.1074/jbc.m111.304477 22094463 PMC3270999

[B112] Lange J. N. Wood K. D. Knight J. Assimos D. G. Holmes R. P. (2012). Glyoxal formation and its role in endogenous oxalate synthesis. *Adv. Urol.* 2012:819202. 10.1155/2012/819202 PMC3332067 22567004

[B113] Lau A. N. Israelsen W. J. Roper J. Sinnamon M. J. Georgeon L. Dayton T. L. (2017). PKM2 is not required for colon cancer initiated by APC loss. *Cancer Metab.* 5:10. 10.1186/s40170-017-0172-1 PMC5707917 29214019

[B114] Le A. Lane A. N. Hamaker M. Bose S. Gouw A. Barbi J. (2012). Glucose-independent glutamine metabolism via TCA cycling for proliferation and survival in B cells. *Cell Metab.* 15 110–121. 10.1016/j.cmet.2011.12.009 22225880 PMC3345194

[B115] Lee I. S. Muragaki Y. Ideguchi T. Hase T. Tsuji M. Ooshima A. (1995). Molecular cloning and sequencing of a cDNA encoding alanine-glyoxylate aminotransferase 2 from rat kidney. *J. Biochem.* 117 856–862. 10.1093/oxfordjournals.jbchem.a124787 7592550

[B116] Leithner K. Hrzenjak A. Trotzmuller M. Moustafa T. Kofeler H. C. Wohlkoenig C. (2015). PCK2 activation mediates an adaptive response to glucose depletion in lung cancer. *Oncogene* 34 1044–1050. 10.1038/onc.2014.47 24632615

[B117] Li X. Peng S. (2013). Identification of metastasis-associated genes in colorectal cancer through an integrated genomic and transcriptomic analysis. *Chin. J. Cancer Res.* 25 623–636. 24385689 10.3978/j.issn.1000-9604.2013.11.01 PMC3872552

[B118] Li Y. H. Li X. F. Liu J. T. Wang H. Fan L. L. Li J. (2018). PKM2, a potential target for regulating cancer. *Gene* 668 48–53. 10.1016/j.gene.2018.05.038 29775756

[B119] Li Z. Yang P. Li Z. (2014). The multifaceted regulation and functions of PKM2 in tumor progression. *Biochim. Biophys. Acta* 1846 285–296. 10.1016/j.bbcan.2014.07.008 25064846

[B120] Lieu E. L. Nguyen T. Rhyne S. Kim J. (2020). Amino acids in cancer. *Exp. Mol. Med.* 52 15–30. 31980738 10.1038/s12276-020-0375-3 PMC7000687

[B121] Liu B. Jia Y. Cao Y. Wu S. Jiang H. Sun X. (2016). Overexpression of Phosphoserine Aminotransferase 1 (PSAT1) Predicts Poor Prognosis and Associates with Tumor Progression in Human Esophageal Squamous Cell Carcinoma. *Cell. Physiol. Biochem.* 39 395–406. 10.1159/000445633 27372650

[B122] Liu C. Cao J. Lin S. Zhao Y. Zhu M. Tao Z. (2020). Malic enzyme 1 indicates worse prognosis in breast cancer and promotes metastasis by manipulating reactive oxygen species. *Onco Targets Ther.* 13 8735–8747. 10.2147/ott.s256970 32922044 PMC7457736

[B123] Liu P. Ge X. Ding H. Jiang H. Christensen B. M. Li J. (2012). Role of glutamate decarboxylase-like protein 1 (GADL1) in taurine biosynthesis. *J. Biol. Chem.* 287 40898–40906. 10.1074/jbc.m112.393728 23038267 PMC3510794

[B124] Liu V. M. Howell A. J. Hosios A. M. Li Z. Israelsen W. J. Vander Heiden M. G. (2020). Cancer-associated mutations in human pyruvate kinase M2 impair enzyme activity. *FEBS Lett.* 594 646–664. 10.1002/1873-3468.13648 31642061 PMC7042059

[B125] Loeber G. Dworkin M. B. Infante A. Ahorn H. (1994). Characterization of cytosolic malic enzyme in human tumor cells. *FEBS Lett.* 344 181–186. 10.1016/0014-5793(94)00386-6 8187880

[B126] Lunt S. Y. Muralidhar V. Hosios A. M. Israelsen W. J. Gui D. Y. Newhouse L. (2015). Pyruvate kinase isoform expression alters nucleotide synthesis to impact cell proliferation. *Mol. Cell* 57 95–107. 10.1016/j.molcel.2014.10.027 25482511 PMC4289430

[B127] Luo W. Hu H. Chang R. Zhong J. Knabel M. O’Meally R. (2011). Pyruvate kinase M2 is a PHD3-stimulated coactivator for hypoxia-inducible factor 1. *Cell* 145 732–744. 10.1016/j.cell.2011.03.054 21620138 PMC3130564

[B128] Lv L. Li D. Zhao D. Lin R. Chu Y. Zhang H. (2011). Acetylation targets the M2 isoform of pyruvate kinase for degradation through chaperone-mediated autophagy and promotes tumor growth. *Mol. Cell* 42 719–730. 10.1016/j.molcel.2011.04.025 21700219 PMC4879880

[B129] Makinen A. L. Nowak T. (1983). 3-Mercaptopicolinate. A reversible active site inhibitor of avian liver phosphoenolpyruvate carboxykinase. *J. Biol. Chem.* 258 11654–11662. 6619135

[B130] Matoba S. Kang J. G. Patino W. D. Wragg A. Boehm M. Gavrilova O. (2006). p53 regulates mitochondrial respiration. *Science* 312 1650–1653. 10.1126/science.1126863 16728594

[B131] Maul D. M. Schuster S. M. (1986). Kinetic properties and characteristics of mouse liver mitochondrial asparagine aminotransferase. *Arch. Biochem. Biophys.* 251 585–593. 10.1016/0003-9861(86)90367-x 3099645

[B132] Mayers J. R. Torrence M. E. Danai L. V. Papagiannakopoulos T. Davidson S. M. Bauer M. R. (2016). Tissue of origin dictates branched-chain amino acid metabolism in mutant Kras-driven cancers. *Science* 353 1161–1165. 10.1126/science.aaf5171 27609895 PMC5245791

[B133] Mazurek S. Boschek C. B. Hugo F. Eigenbrodt E. (2005). Pyruvate kinase type M2 and its role in tumor growth and spreading. *Semin. Cancer Biol.* 15 300–308. 10.1016/j.semcancer.2005.04.009 15908230

[B134] McKeehan W. L. (1982). Glycolysis, glutaminolysis and cell proliferation. *Cell Biol. Int. Rep.* 6 635–650. 10.1016/0309-1651(82)90125-4 6751566

[B135] Mdluli K. Booth M. P. Brady R. L. Rumsby G. (2005). A preliminary account of the properties of recombinant human Glyoxylate reductase (GRHPR), LDHA and LDHB with glyoxylate, and their potential roles in its metabolism. *Biochim. Biophys. Acta* 1753 209–216. 10.1016/j.bbapap.2005.08.004 16198644

[B136] Medina D. Thompson H. Ganther H. Ip C. (2001). Se-methylselenocysteine: a new compound for chemoprevention of breast cancer. *Nutr. Cancer* 40 12–17. 10.4324/9781410608000-4 11799917

[B137] Metcalf S. Dougherty S. Kruer T. Hasan N. Biyik-Sit R. Reynolds L. (2020). Selective loss of phosphoserine aminotransferase 1 (PSAT1) suppresses migration, invasion, and experimental metastasis in triple negative breast cancer. *Clin. Exp. Metastasis* 37 187–197. 10.1007/s10585-019-10000-7 31630284

[B138] Michelakis E. D. Webster L. Mackey J. R. (2008). Dichloroacetate (DCA) as a potential metabolic-targeting therapy for cancer. *Br. J. Cancer* 99 989–994. 10.1038/sj.bjc.6604554 18766181 PMC2567082

[B139] Mills E. L. Kelly B. Logan A. Costa A. S. H. Varma M. Bryant C. E. (2016). Succinate Dehydrogenase supports metabolic repurposing of mitochondria to drive inflammatory macrophages. *Cell* 167 457–470.e13. 27667687 10.1016/j.cell.2016.08.064 PMC5863951

[B140] Minatogawa Y. Noguchi T. Kido R. (1977). Species distribution and properties of hepatic phenylalanine (histidine):pyruvate aminotransferase. *Hoppe Seylers Z. Physiol. Chem.* 358 59–67. 10.1515/bchm2.1977.358.1.59 14070

[B141] Moreno-Sanchez R. Marin-Hernandez A. Gallardo-Perez J. C. Pacheco-Velazquez S. C. Robledo-Cadena D. X. Padilla-Flores J. A. (2020). Physiological Role of Glutamate Dehydrogenase in Cancer Cells. *Front. Oncol.* 10:429. 10.3389/fonc.2020.00429 32328457 PMC7160333

[B142] Moretti S. Martin O. Van Du Tran T. Bridge A. Morgat A. Pagni M. (2016). MetaNetX/MNXref–reconciliation of metabolites and biochemical reactions to bring together genome-scale metabolic networks. *Nucleic Acids Res.* 44 D523–D526. 26527720 10.1093/nar/gkv1117 PMC4702813

[B143] Mose S. Menzel C. Kurth A. A. Obert K. Breidert I. Borowsky K. (2003). Tartrate-resistant acid phosphatase 5b as serum marker of bone metabolism in cancer patients. *Anticancer Res.* 23 2783–2788. 12926113

[B144] Murai S. Ando A. Ebara S. Hirayama M. Satomi Y. Hara T. (2017). Inhibition of malic enzyme 1 disrupts cellular metabolism and leads to vulnerability in cancer cells in glucose-restricted conditions. *Oncogenesis* 6:e329. 10.1038/oncsis.2017.34 28481367 PMC5523067

[B145] Murata M. Midorikawa K. Kawanishi S. (2016). “Chapter 25 - Molecular Link Between Alcohol and Breast Cancer: the Role of Salsolinol,” in *Molecular Aspects of Alcohol and Nutrition*, ed. Patel V. B. (San Diego, CA: Academic Press), 315–324. 10.1016/b978-0-12-800773-0.00025-2

[B146] Nagahara N. Sawada N. (2006). The mercaptopyruvate pathway in cysteine catabolism: a physiologic role and related disease of the multifunctional 3-mercaptopyruvate sulfurtransferase. *Curr. Med. Chem.* 13 1219–1230. 10.2174/092986706776360914 16719781

[B147] Nagasawa T. Ishii T. Kumagai H. Yamada H. (1985). D-Cysteine desulfhydrase of *Escherichia coli*. Purification and characterization. *Eur. J. Biochem.* 153 541–551. 10.1111/j.1432-1033.1985.tb09335.x 3908101

[B148] Nagata Y. Masui R. Akino T. (1992). The presence of free D-serine, D-alanine and D-proline in human plasma. *Experientia* 48 986–988. 1426150 10.1007/BF01919147

[B149] Nagayama H. Muramatsu M. Shimura K. (1958). Enzymatic formation of aminomalonic acid from ketomalonic acid. *Nature* 181 417–418. 10.1038/181417a0 13504217

[B150] Nakano M. Tsutsumi Y. Danowski T. S. (1967). Crystalline L-amino-acid oxidase from the soluble fraction of rat-kidney cells. *Biochim. Biophys. Acta* 139 40–48. 10.1016/0005-2744(67)90111-8 4962138

[B151] Nakashima C. Kirita T. Yamamoto K. Mori S. Luo Y. Sasaki T. (2020). Malic enzyme 1 is associated with tumor budding in oral squamous cell carcinomas. *Int. J. Mol. Sci.* 21:7149. 10.3390/ijms21197149 32998265 PMC7582746

[B152] Nemeth B. Doczi J. Csete D. Kacso G. Ravasz D. Adams D. (2016). Abolition of mitochondrial substrate-level phosphorylation by itaconic acid produced by LPS-induced Irg1 expression in cells of murine macrophage lineage. *FASEB J.* 30 286–300. 10.1096/fj.15-279398 26358042

[B153] Newman A. C. Maddocks O. D. K. (2017). Serine and functional metabolites in cancer. *Trends Cell Biol.* 27 645–657. 10.1016/j.tcb.2017.05.001 28601431

[B154] Nguyen M. Bonneterre J. Hecquet B. Desoize B. Demaille A. (1991). Plasma acid and alkaline phosphatase in patients with breast cancer. *Anticancer Res.* 11 831–833. 2064338

[B155] Nicolay B. N. Danielian P. S. Kottakis F. Lapek J. D. Jr. Sanidas I. Miles W. O. (2015). Proteomic analysis of pRb loss highlights a signature of decreased mitochondrial oxidative phosphorylation. *Genes Dev.* 29 1875–1889. 10.1101/gad.264127.115 26314710 PMC4573859

[B156] Nokin M. J. Bellier J. Durieux F. Peulen O. Rademaker G. Gabriel M. (2019). Methylglyoxal, a glycolysis metabolite, triggers metastasis through MEK/ERK/SMAD1 pathway activation in breast cancer. *Breast Cancer Res.* 21:11. 10.1186/s13058-018-1095-7 PMC6343302 30674353

[B157] Nordlie R. C. (1974). Metabolic regulation by multifunctional glucose-6-phosphatase. *Curr. Top. Cell. Regul.* 8 33–117. 10.1016/b978-0-12-152808-9.50009-2 4370737

[B158] Noronha A. Modamio J. Jarosz Y. Guerard E. Sompairac N. Preciat G. (2019). The Virtual Metabolic Human database: integrating human and gut microbiome metabolism with nutrition and disease. *Nucleic Acids Res.* 47 D614–D624. 30371894 10.1093/nar/gky992 PMC6323901

[B159] Ogawa H. Gomi T. Nishizawa M. Hayakawa Y. Endo S. Hayashi K. (2006). Enzymatic and biochemical properties of a novel human serine dehydratase isoform. *Biochim. Biophys. Acta* 1764 961–971. 10.1016/j.bbapap.2006.02.010 16580895

[B160] Omura H. Shimamura M. Yamafuji K. (1956). Measurement of transoximase action. *Enzymologia* 17 359–362. 13397521

[B161] Orywal K. Szmitkowski M. (2017). Alcohol dehydrogenase and aldehyde dehydrogenase in malignant neoplasms. *Clin. Exp. Med.* 17 131–139. 10.1007/s10238-016-0408-3 26886278 PMC5403859

[B162] Pallini R. Guazzi G. C. Cannella C. Cacace M. G. (1991). Cloning and sequence analysis of the human liver rhodanese: comparison with the bovine and chicken enzymes. *Biochem. Biophys. Res. Commun.* 180 887–893. 10.1016/s0006-291x(05)81148-9 1953758

[B163] Panosyan E. H. Wang Y. Xia P. Lee W. N. Pak Y. Laks D. R. (2014). Asparagine depletion potentiates the cytotoxic effect of chemotherapy against brain tumors. *Mol. Cancer Res.* 12 694–702. 10.1158/1541-7786.mcr-13-0576 24505127 PMC4020976

[B164] Paone A. Marani M. Fiascarelli A. Rinaldo S. Giardina G. Contestabile R. (2014). SHMT1 knockdown induces apoptosis in lung cancer cells by causing uracil misincorporation. *Cell Death Dis.* 5:e1525. 10.1038/cddis.2014.482 25412303 PMC4260740

[B165] Passarella S. Atlante A. Valenti D. de Bari L. (2003). The role of mitochondrial transport in energy metabolism. *Mitochondrion* 2 319–343. 10.1016/s1567-7249(03)00008-4 16120331

[B166] Pastuszak I. Drake R. Elbein A. D. (1996). Kidney N-acetylgalactosamine (GalNAc)-1-phosphate kinase, a new pathway of GalNAc activation. *J. Biol. Chem.* 271 20776–20782. 10.1074/jbc.271.34.20776 8702831

[B167] Petit M. Koziel R. Etemad S. Pircher H. Jansen-Durr P. (2017). Depletion of oxaloacetate decarboxylase FAHD1 inhibits mitochondrial electron transport and induces cellular senescence in human endothelial cells. *Exp. Gerontol.* 92 7–12. 10.1016/j.exger.2017.03.004 28286170

[B168] Phang J. M. (2019). Proline metabolism in cell regulation and cancer biology: recent advances and hypotheses. *Antioxid. Redox Signal.* 30 635–649. 10.1089/ars.2017.7350 28990419 PMC6338564

[B169] Pinthong C. Maenpuen S. Amornwatcharapong W. Yuthavong Y. Leartsakulpanich U. Chaiyen P. (2014). Distinct biochemical properties of human serine hydroxymethyltransferase compared with the Plasmodium enzyme: implications for selective inhibition. *FEBS J.* 281 2570–2583. 10.1111/febs.12803 24698160

[B170] Pircher H. Straganz G. D. Ehehalt D. Morrow G. Tanguay R. M. Jansen-Durr P. (2011). Identification of human fumarylacetoacetate hydrolase domain-containing protein 1 (FAHD1) as a novel mitochondrial acylpyruvase. *J. Biol. Chem.* 286 36500–36508. 10.1074/jbc.m111.264770 21878618 PMC3196145

[B171] Pircher H. von Grafenstein S. Diener T. Metzger C. Albertini E. Taferner A. (2015). Identification of FAH domain-containing protein 1 (FAHD1) as oxaloacetate decarboxylase. *J. Biol. Chem.* 290 6755–6762. 10.1074/jbc.m114.609305 25575590 PMC4358102

[B172] Poore R. E. Hurst C. H. Assimos D. G. Holmes R. P. (1997). Pathways of hepatic oxalate synthesis and their regulation. *Am. J. Physiol.* 272 C289–C294. 9038835 10.1152/ajpcell.1997.272.1.C289

[B173] Prakasam G. Iqbal M. A. Bamezai R. N. K. Mazurek S. (2018). Posttranslational Modifications of Pyruvate Kinase M2: tweaks that benefit cancer. *Front. Oncol.* 8:22. 10.3389/fonc.2018.00022 29468140 PMC5808394

[B174] Reitzer L. J. Wice B. M. Kennell D. (1979). Evidence that glutamine, not sugar, is the major energy source for cultured HeLa cells. *J. Biol. Chem.* 254 2669–2676. 429309

[B175] Ren J. G. Seth P. Clish C. B. Lorkiewicz P. K. Higashi R. M. Lane A. N. (2014). Knockdown of malic enzyme 2 suppresses lung tumor growth, induces differentiation and impacts PI3K/AKT signaling. *Sci. Rep.* 4:5414. 10.1038/srep05414 PMC4067620 24957098

[B176] Richarme G. Dairou J. (2017). Parkinsonism-associated protein DJ-1 is a bona fide deglycase. *Biochem. Biophys. Res. Commun.* 483 387–391. 10.1016/j.bbrc.2016.12.134 28013050

[B177] Richarme G. Mihoub M. Dairou J. Bui L. C. Leger T. Lamouri A. (2015). Parkinsonism-associated protein DJ-1/Park7 is a major protein deglycase that repairs methylglyoxal- and glyoxal-glycated cysteine, arginine, and lysine residues. *J. Biol. Chem.* 290 1885–1897. 10.1074/jbc.m114.597815 25416785 PMC4340429

[B178] Robinson B. H. (1971). Transport of phosphoenolpyruvate by the tricarboxylate transporting system in mammalian mitochondria. *FEBS Lett.* 14 309–312. 10.1016/0014-5793(71)80287-9 11945784

[B179] Robinson J. L. Kocabas P. Wang H. Cholley P. E. Cook D. Nilsson A. (2020). An atlas of human metabolism. *Sci. Signal.* 13:eaaz1482. 10.1126/scisignal.aaz1482 PMC7331181 32209698

[B180] Rowsell E. V. (1956). Transaminations with pyruvate and other alpha-keto acids. *Biochem. J.* 64 246–252. 10.1042/bj0640246 13363834 PMC1199724

[B181] Sandler M. Carter S. B. Hunter K. R. Stern G. M. (1973). Tetrahydroisoquinoline alkaloids: in vivo metabolites of L-dopa in man. *Nature* 241 439–443. 10.1038/241439a0 4705752

[B182] Satrustegui J. Pardo B. Del Arco A. (2007). Mitochondrial transporters as novel targets for intracellular calcium signaling. *Physiol. Rev.* 87 29–67. 10.1152/physrev.00005.2006 17237342

[B183] Sauer L. A. (1973). An NAD- and NADP-dependent malic enzyme with regulatory properties in rat liver and adrenal cortex mitochondrial fractions. *Biochem. Biophys. Res. Commun.* 50 524–531. 10.1016/0006-291x(73)90871-1 4144006

[B184] Schadewaldt P. Wendel U. Hammen H. W. (1995). Human branched-chain L-amino acid aminotransferase: activity and subcellular localization in cultured skin fibroblasts. *Amino Acids* 9 147–160. 24178815 10.1007/BF00805836

[B185] Scott D. A. Richardson A. D. Filipp F. V. Knutzen C. A. Chiang G. G. Ronai Z. A. (2011). Comparative metabolic flux profiling of melanoma cell lines: beyond the Warburg effect. *J. Biol. Chem.* 286 42626–42634. 10.1074/jbc.m111.282046 21998308 PMC3234981

[B186] Serpa J. (2020). Cysteine as a Carbon Source, a Hot Spot in Cancer Cells Survival. *Front. Oncol.* 10:947. 10.3389/fonc.2020.00947 32714858 PMC7344258

[B187] Shan Y. Gao Y. Jin W. Fan M. Wang Y. Gu Y. (2019). Targeting HIBCH to reprogram valine metabolism for the treatment of colorectal cancer. *Cell Death Dis.* 10 618. 10.1038/s41419-019-1832-6 PMC6692300 31409769

[B188] Shapiro R. A. Clark V. M. Curthoys N. P. (1979). Inactivation of rat renal phosphate-dependent glutaminase with 6-diazo-5-oxo-L-norleucine. Evidence for interaction at the glutamine binding site. *J. Biol. Chem.* 254 2835–2838. 429321

[B189] Shen H. Campanello G. C. Flicker D. Grabarek Z. Hu J. Luo C. (2017). The human knockout gene CLYBL connects Itaconate to Vitamin B12. *Cell* 171 771–782.e11. 29056341 10.1016/j.cell.2017.09.051 PMC5827971

[B190] Shibuya N. Koike S. Tanaka M. Ishigami-Yuasa M. Kimura Y. Ogasawara Y. (2013). A novel pathway for the production of hydrogen sulfide from D-cysteine in mammalian cells. *Nat. Commun.* 4:1366. 10.1038/ncomms2371 23340406

[B191] Shug A. L. Shrago E. (1973). Inhibition of phosphoenolpyruvate transport via the tricarboxylate and adenine nucleotide carrier systems of rat liver mitochondria. *Biochem. Biophys. Res. Commun.* 53 659–665. 10.1016/0006-291x(73)90712-2 4716993

[B192] Silverstein E. (1974). Equilibrium kinetic study of bovine liver glutamate dehydrogenase at high pH. *Biochemistry* 13 3750–3754. 10.1021/bi00715a021 4368692

[B193] Smith A. L. Whitehall J. C. Bradshaw C. Gay D. Robertson F. Blain A. P. (2020). Age-associated mitochondrial DNA mutations cause metabolic remodelling that contributes to accelerated intestinal tumorigenesis. *Nat. Cancer* 1 976–989. 10.1038/s43018-020-00112-5 33073241 PMC7116185

[B194] Smith S. M. Uslaner J. M. Yao L. Mullins C. M. Surles N. O. Huszar S. L. (2009). The behavioral and neurochemical effects of a novel D-amino acid oxidase inhibitor compound 8 [4H-thieno [3,2-b]pyrrole-5-carboxylic acid] and D-serine. *J. Pharmacol. Exp. Ther.* 328 921–930. 10.1124/jpet.108.147884 19088300

[B195] Snell K. Natsumeda Y. Eble J. N. Glover J. L. Weber G. (1988). Enzymic imbalance in serine metabolism in human colon carcinoma and rat sarcoma. *Br. J. Cancer* 57 87–90. 10.1038/bjc.1988.15 3126791 PMC2246686

[B196] Soda K. Osumi T. (1969). Crystalline amino acid racemase with low substrate specificity. *Biochem. Biophys. Res. Commun.* 35 363–368. 10.1016/0006-291x(69)90507-5 5788493

[B197] Soling H. D. Walter U. Sauer H. Kleineke J. (1971). Effects of synthetic analogues of phosphoenolpyruvate on muscle and liver pyruvate kinase, muscle enolase, liver phosphoenolpyruvate carboxykinase and on the intra-/extra-mitochondrial tricarboxylic acid carrier transport system. *FEBS Lett.* 19 139–143. 10.1016/0014-5793(71)80498-2 11946196

[B198] Son J. Lyssiotis C. A. Ying H. Wang X. Hua S. Ligorio M. (2013). Glutamine supports pancreatic cancer growth through a KRAS-regulated metabolic pathway. *Nature* 496 101–105. 10.1038/nature12040 23535601 PMC3656466

[B199] Stark R. Pasquel F. Turcu A. Pongratz R. L. Roden M. Cline G. W. (2009). Phosphoenolpyruvate cycling via mitochondrial phosphoenolpyruvate carboxykinase links anaplerosis and mitochondrial GTP with insulin secretion. *J. Biol. Chem.* 284 26578–26590. 10.1074/jbc.m109.011775 19635791 PMC2785346

[B200] Stetak A. Veress R. Ovadi J. Csermely P. Keri G. Ullrich A. (2007). Nuclear translocation of the tumor marker pyruvate kinase M2 induces programmed cell death. *Cancer Res.* 67 1602–1608. 10.1158/0008-5472.can-06-2870 17308100

[B201] Stipanuk M. H. (1979). Effect of excess dietary methionine on the catabolism of cysteine in rats. *J. Nutr.* 109 2126–2139. 10.1093/jn/109.12.2126 512701

[B202] Stipanuk M. H. (2020). Metabolism of Sulfur-Containing Amino Acids: how the Body Copes with Excess Methionine, Cysteine, and Sulfide. *J. Nutr.* 150(Suppl. 1), 2494S–2505S. 33000151 10.1093/jn/nxaa094

[B203] Stipanuk M. H. Dominy J. E. Jr. Lee J. I. Coloso R. M. (2006). Mammalian cysteine metabolism: new insights into regulation of cysteine metabolism. *J. Nutr.* 136(6 Suppl.), 1652S–1659S. 16702335 10.1093/jn/136.6.1652S

[B204] Sul H. S. Shrago E. Shug A. L. (1976). Relationship of phosphoenolpyruvate transport, acyl coenzyme A inhibition of adenine nucleotide translocase and calcium ion efflux in guinea pig heart mitochondria. *Arch. Biochem. Biophys.* 172 230–237. 10.1016/0003-9861(76)90071-0 1252077

[B205] Sun Y. Li W. Shen S. Yang X. Lu B. Zhang X. (2019). Loss of alanine-glyoxylate and serine-pyruvate aminotransferase expression accelerated the progression of hepatocellular carcinoma and predicted poor prognosis. *J. Transl. Med.* 17:390. 10.1186/s12967-019-02138-5 PMC6880547 31771612

[B206] Swick R. W. Wood H. G. (1960). The Role of Transcarboxylation in Propionic Acid Fermentation. *Proc. Natl. Acad. Sci. U.S.A.* 46 28–41. 10.1073/pnas.46.1.28 16590594 PMC285006

[B207] Ta N. L. Seyfried T. N. (2015). Influence of Serum and Hypoxia on Incorporation of [(14)C]-D-Glucose or [(14)C]-L-Glutamine into Lipids and Lactate in Murine Glioblastoma Cells. *Lipids* 50 1167–1184. 10.1007/s11745-015-4075-z 26537505

[B208] Tech K. Tikunov A. P. Farooq H. Morrissy A. S. Meidinger J. Fish T. (2017). Pyruvate Kinase Inhibits Proliferation during Postnatal Cerebellar Neurogenesis and Suppresses Medulloblastoma Formation. *Cancer Res.* 77 3217–3230. 10.1158/0008-5472.can-16-3304 28515149 PMC5497486

[B209] Terpos E. de la Fuente J. Szydlo R. Hatjiharissi E. Viniou N. Meletis J. (2003). Tartrate-resistant acid phosphatase isoform 5b: a novel serum marker for monitoring bone disease in multiple myeloma. *Int. J. Cancer* 106 455–457. 10.1002/ijc.11247 12845688

[B210] Thakur A. Bollig A. Wu J. Liao D. J. (2008). Gene expression profiles in primary pancreatic tumors and metastatic lesions of Ela-c-myc transgenic mice. *Mol. Cancer* 7:11. 10.1186/1476-4598-7-11 18218118 PMC2259361

[B211] Thornalley P. J. (1990). The glyoxalase system: new developments towards functional characterization of a metabolic pathway fundamental to biological life. *Biochem. J.* 269 1–11. 10.1042/bj2690001 2198020 PMC1131522

[B212] Tsun Z. Y. Possemato R. (2015). Amino acid management in cancer. *Semin. Cell Dev. Biol.* 43 22–32. 26277542 10.1016/j.semcdb.2015.08.002 PMC4800996

[B213] Tudball N. Thomas P. (1972). The enzymic degradation of L-serine O-sulphate, Mechanism of the reaction. *Biochem. J.* 128 41–46. 10.1042/bj1280041 4673572 PMC1173567

[B214] Vander Heiden M. G. Cantley L. C. Thompson C. B. (2009). Understanding the Warburg effect: the metabolic requirements of cell proliferation. *Science* 324 1029–1033. 10.1126/science.1160809 19460998 PMC2849637

[B215] Vander Heiden M. G. Locasale J. W. Swanson K. D. Sharfi H. Heffron G. J. Amador-Noguez D. (2010). Evidence for an alternative glycolytic pathway in rapidly proliferating cells. *Science* 329 1492–1499. 10.1126/science.1188015 20847263 PMC3030121

[B216] Vander Jagt D. L. Hunsaker L. A. (2003). Methylglyoxal metabolism and diabetic complications: roles of aldose reductase, glyoxalase-I, betaine aldehyde dehydrogenase and 2-oxoaldehyde dehydrogenase. *Chem. Biol. Interact.* 14 341–351. 10.1016/s0009-2797(02)00212-0 12604221

[B217] Vasseur S. Afzal S. Tardivel-Lacombe J. Park D. S. Iovanna J. L. Mak T. W. (2009). DJ-1/PARK7 is an important mediator of hypoxia-induced cellular responses. *Proc. Natl. Acad. Sci. U.S.A.* 106 1111–1116. 10.1073/pnas.0812745106 19144925 PMC2626605

[B218] Vignaud C. Pietrancosta N. Williams E. L. Rumsby G. Lederer F. (2007). Purification and characterization of recombinant human liver glycolate oxidase. *Arch. Biochem. Biophys.* 465 410–416. 10.1016/j.abb.2007.06.021 17669354

[B219] Vincent E. E. Sergushichev A. Griss T. Gingras M. C. Samborska B. Ntimbane T. (2015). Mitochondrial phosphoenolpyruvate carboxykinase regulates metabolic adaptation and enables glucose-independent tumor growth. *Mol. Cell* 60 195–207. 10.1016/j.molcel.2015.08.013 26474064

[B220] Wang Y. H. Huang J. T. Chen W. L. Wang R. H. Kao M. C. Pan Y. R. (2019). Dysregulation of cystathionine gamma-lyase promotes prostate cancer progression and metastasis. *EMBO Rep.* 20:e45986. 10.15252/embr.201845986 PMC6776913 31468690

[B221] Wang Y. H. Israelsen W. J. Lee D. Yu V. W. C. Jeanson N. T. Clish C. B. (2014). Cell-state-specific metabolic dependency in hematopoiesis and leukemogenesis. *Cell* 158 1309–1323. 10.1016/j.cell.2014.07.048 25215489 PMC4197056

[B222] Warren L. (1986). Sialic acid lyase in human promyelocytic leukemic cells (HL-60) during phorbol-ester-induced differentiation. *Biochim. Biophys. Acta* 888 278–281. 10.1016/0167-4889(86)90226-0 3463364

[B223] Weber G. (1969). Inhibition of human brain pyruvate kinase and hexokinase by phenylalanine and phenylpyruvate: possible relevance to phenylketonuric brain damage. *Proc. Natl. Acad. Sci. U.S.A.* 63 1365–1369. 10.1073/pnas.63.4.1365 5260939 PMC223473

[B224] Weber G. Cantero A. (1955). Glucose-6-phosphatase activity in normal, pre-cancerous, and neoplastic tissues. *Cancer Res.* 15 105–108. 14352196

[B225] Wiese T. J. Wuensch S. A. Ray P. D. (1996). Synthesis of citrate from phosphoenolpyruvate and acetylcarnitine by mitochondria from rabbit, pigeon and rat liver: implications for lipogenesis. *Comp. Biochem. Physiol. B Biochem. Mol. Biol.* 114 417–422. 10.1016/0305-0491(96)00035-1 8840517

[B226] Wilson D. F. Erecinska M. Schramm V. L. (1983). Evaluation of the relationship between the intra- and extramitochondrial [ATP]/[ADP] ratios using phosphoenolpyruvate carboxykinase. *J. Biol. Chem.* 258 10464–10473. 6885788

[B227] Windmueller H. G. Spaeth A. E. (1974). Uptake and metabolism of plasma glutamine by the small intestine. *J. Biol. Chem.* 249 5070–5079. 4605420

[B228] Wishart D. S. Feunang Y. D. Marcu A. Guo A. C. Liang K. Vazquez-Fresno R. (2018). HMDB 4.0: the human metabolome database for 2018. *Nucleic Acids Res.* 46 D608–D617. 29140435 10.1093/nar/gkx1089 PMC5753273

[B229] Wong N. Ojo D. Yan J. Tang D. (2015). PKM2 contributes to cancer metabolism. *Cancer Lett.* 356 184–191. 10.1016/j.canlet.2014.01.031 24508027

[B230] Wuensch S. A. Ray P. D. (1997). Synthesis of citrate from phosphoenolpyruvate and acetylcarnitine by mitochondria from rabbit enterocytes: implications for lipogenesis. *Comp. Biochem. Physiol. B Biochem. Mol. Biol.* 118 599–605. 10.1016/s0305-0491(97)00242-3 9467872

[B231] Yamada K. Noguchi T. (1999). Nutrient and hormonal regulation of pyruvate kinase gene expression. *Biochem. J.* 337(Pt 1), 1–11. 10.1042/0264-6021:3370001 9854017 PMC1219928

[B232] Yang H. Jia X. (2014). Safety evaluation of Se-methylselenocysteine as nutritional selenium supplement: acute toxicity, genotoxicity and subchronic toxicity. *Regul. Toxicol. Pharmacol.* 70 720–727. 10.1016/j.yrtph.2014.10.014 25444999

[B233] Yang M. Vousden K. H. (2016). Serine and one-carbon metabolism in cancer. *Nat. Rev. Cancer* 16 650–662. 10.1038/nrc.2016.81 27634448

[B234] Yang W. Lu Z. (2013). Nuclear PKM2 regulates the Warburg effect. *Cell Cycle* 12 3154–3158. 24013426 10.4161/cc.26182 PMC3865010

[B235] Yang W. Lu Z. (2015). Pyruvate kinase M2 at a glance. *J. Cell Sci.* 128 1655–1660. 10.1242/jcs.166629 25770102 PMC4446733

[B236] Yang W. Zheng Y. Xia Y. Ji H. Chen X. Guo F. (2012). ERK1/2-dependent phosphorylation and nuclear translocation of PKM2 promotes the Warburg effect. *Nat. Cell Biol.* 14 1295–1304. 10.1038/ncb2629 23178880 PMC3511602

[B237] Ye D. Guan K. L. Xiong Y. (2018). Metabolism, Activity, and Targeting of D- and L-2-Hydroxyglutarates. *Trends Cancer* 4 151–165. 10.1016/j.trecan.2017.12.005 29458964 PMC5884165

[B238] Yoon S. Kim J. G. Seo A. N. Park S. Y. Kim H. J. Park J. S. (2015). Clinical implication of serine metabolism-associated enzymes in colon cancer. *Oncology* 89 351–359. 10.1159/000439571 26439504

[B239] You J. Shi X. Liang H. Ye J. Wang L. Han H. (2017). Cystathionine- gamma-lyase promotes process of breast cancer in association with STAT3 signaling pathway. *Oncotarget* 8 65677–65686. 10.18632/oncotarget.20057 29029463 PMC5630363

[B240] Yu L. Teoh S. T. Ensink E. Ogrodzinski M. P. Yang C. Vazquez A. I. (2019). Cysteine catabolism and the serine biosynthesis pathway support pyruvate production during pyruvate kinase knockdown in pancreatic cancer cells. *Cancer Metab.* 7:13. 10.1186/s40170-019-0205-z PMC6937848 31893043

[B241] Yu Y. H. Chang Y. C. Su T. H. Nong J. Y. Li C. C. Chuang L. M. (2013). Prostaglandin reductase-3 negatively modulates adipogenesis through regulation of PPARgamma activity. *J. Lipid Res.* 54 2391–2399. 10.1194/jlr.m037556 23821743 PMC3735937

[B242] Yuan M. McNae I. W. Chen Y. Blackburn E. A. Wear M. A. Michels P. A. M. (2018). An allostatic mechanism for M2 pyruvate kinase as an amino-acid sensor. *Biochem. J.* 475 1821–1837. 10.1042/bcj20180171 29748232 PMC5980995

[B243] Yuen C. A. Asuthkar S. Guda M. R. Tsung A. J. Velpula K. K. (2016). Cancer stem cell molecular reprogramming of the Warburg effect in glioblastomas: a new target gleaned from an old concept. *CNS Oncol.* 5 101–108. 10.2217/cns-2015-0006 26997129 PMC6047435

[B244] Zeidan Q. Hart G. W. (2010). The intersections between O-GlcNAcylation and phosphorylation: implications for multiple signaling pathways. *J. Cell Sci.* 123 13–22. 10.1242/jcs.053678 20016062 PMC2794709

[B245] Zelewski M. Swierczynski J. (1991). Malic enzyme in human liver. Intracellular distribution, purification and properties of cytosolic isozyme. *Eur. J. Biochem.* 201 339–345. 10.1111/j.1432-1033.1991.tb16291.x 1935931

[B246] Zhang Z. Deng X. Liu Y. Liu Y. Sun L. Chen F. (2019). PKM2, function and expression and regulation. *Cell Biosci.* 9:52. 10.1186/s13578-019-0317-8 PMC6595688 31391918

[B247] Zhao J. Li J. Fan T. W. M. Hou S. X. (2017). Glycolytic reprogramming through PCK2 regulates tumor initiation of prostate cancer cells. *Oncotarget* 8 83602–83618. 10.18632/oncotarget.18787 29137367 PMC5663539

[B248] Zhou H. L. Zhang R. Anand P. Stomberski C. T. Qian Z. Hausladen A. (2019). Metabolic reprogramming by the S-nitroso-CoA reductase system protects against kidney injury. *Nature* 565 96–100. 10.1038/s41586-018-0749-z 30487609 PMC6318002

[B249] Zhou S. Kachhap S. Singh K. K. (2003). Mitochondrial impairment in p53-deficient human cancer cells. *Mutagenesis* 18 287–292. 10.1093/mutage/18.3.287 12714696

[B250] Zielke H. R. Sumbilla C. M. Sevdalian D. A. Hawkins R. L. Ozand P. T. (1980). Lactate: a major product of glutamine metabolism by human diploid fibroblasts. *J. Cell. Physiol.* 104 433–441. 10.1002/jcp.1041040316 7419614

